# Mitochondrial-Centered Biological Networks in Metabolic Disease: Toward Precision Mitochondrial Medicine

**DOI:** 10.3390/ijms27167334

**Published:** 2026-08-17

**Authors:** Victoriano Pérez-Vázquez, Juan Manuel Guzmán-Flores, Katya Vargas-Ortiz, Carmen Palacios-Reyes, Joel Ramírez-Emiliano

**Affiliations:** 1 Department of Medical Sciences, University of Guanajuato, Campus León, León 37320, Guanajuato, Mexico; k.vargasortiz@ugto.mx (K.V.-O.); mdc.palacios@ugto.mx (C.P.-R.); joelre@ugto.mx (J.R.-E.); 2 Instituto de Investigación en Biociencias, Centro Universitario de los Altos, Universidad de Guadalajara, Tepatitlán de Morelos 47620, Jalisco, Mexico; jmanuel.guzman@academicos.udg.mx

**Keywords:** obesity, type 2 diabetes, comparative proteomics, mitochondrial homeostasis, mitochondrial-centered biological networks, systems biology, network medicine, curcumin, precision mitochondrial medicine

## Abstract

Obesity and type 2 diabetes (T2D) are multifactorial metabolic disorders characterized by progressive dysfunction of multiple organs and biological systems. Although mitochondrial dysfunction is a hallmark of disease progression, the mechanisms linking metabolic stress to coordinated tissue dysfunction remain incompletely understood. Comparative proteomic studies have consistently identified coordinated remodeling of oxidative phosphorylation, fatty acid oxidation, tricarboxylic acid cycle activity, redox regulation, mitochondrial proteostasis, and adaptive signaling across metabolically affected organs, revealing conserved organizational principles underlying mitochondrial adaptation. However, these findings have largely been interpreted within reductionist, pathway-centered frameworks. Here, we integrate evidence from comparative proteomics, mitochondrial biology, bioenergetics, redox biology, signaling, and systems biology to propose the concept of mitochondrial-centered biological networks (MCBNs), in which mitochondria function as dynamic regulatory hubs coordinating interconnected processes that collectively determine metabolic adaptation and tissue resilience. Building on this framework, we introduce the Mitochondrial Homeostasis Hypothesis, which proposes that preservation or restoration of mitochondrial homeostasis depends on coordinated regulation of MCBNs and constitutes a fundamental systems-level mechanism underlying resistance to obesity, T2D, and hypercaloric diet-induced metabolic dysfunction. Curcumin represents a well-studied network-modulating intervention that coordinately influences mitochondrial bioenergetics, metabolic flexibility, redox homeostasis, proteostasis, inflammatory signaling, and adaptive stress responses, supporting the concept that mitochondrial homeostasis is preserved through coordinated network regulation rather than isolated modulation of individual molecular pathways. Finally, we discuss how emerging technologies, including functional proteomics, redox proteomics, spatial and single-cell proteomics, acetylomics, integrated multi-omics, and artificial intelligence-assisted network analysis, provide unprecedented opportunities to quantitatively characterize MCBNs, validate the proposed hypothesis, identify network-based biomarkers, and accelerate the development of network-guided precision mitochondrial medicine.

## 1. Clinical Relevance

### 1.1. The Growing Burden of Metabolic Diseases

Obesity and type 2 diabetes (T2D) have emerged as two of the most important public health challenges worldwide. The prevalence of both conditions has increased dramatically over recent decades, affecting hundreds of millions of individuals and contributing substantially to morbidity, mortality, and healthcare expenditures [1,2]. Beyond their direct metabolic consequences, obesity and T2D significantly increase the risk of cardiovascular disease, chronic kidney disease, metabolic dysfunction-associated steatotic liver disease, neurodegeneration, and several forms of cancer [3,4].

Current therapeutic approaches have achieved important advances in the management of hyperglycemia, dyslipidemia and cardiovascular risk. However, despite the availability of increasingly effective pharmacological interventions, the incidence of metabolic complications remains high, and many patients continue to develop progressive organ dysfunction [5,6]. These observations suggest that important upstream biological mechanisms responsible for coordinating metabolic adaptation remain insufficiently addressed by current treatment strategies.

Increasing evidence indicates that obesity and T2D cannot be fully understood as disorders affecting isolated molecular pathways. Rather, they involve progressive disruption of highly interconnected biological processes governing energy metabolism, redox regulation, inflammatory signaling, and tissue adaptation. This systems-level perspective has stimulated growing interest in identifying integrative mechanisms capable of coordinating multiple aspects of metabolic disease progression simultaneously.

### 1.2. Why Mitochondrial Dysfunction Matters Clinically

Among the mechanisms implicated in metabolic disease progression, mitochondrial dysfunction has emerged as a particularly important candidate. Mitochondria play central roles in cellular energy production, substrate utilization, redox regulation, apoptosis, protein quality control, and adaptive stress responses. As a result, disturbances affecting mitochondrial function have consequences that extend far beyond ATP synthesis alone [7,8].

Accumulating evidence indicates that alterations in oxidative phosphorylation (OXPHOS), fatty acid oxidation (FAO), mitochondrial proteostasis and redox homeostasis occur early during obesity and T2D progression and frequently precede overt tissue injury [3,9]. Mitochondrial dysfunction contributes to insulin resistance, oxidative stress, chronic inflammation, metabolic inflexibility, and fibrotic remodeling, collectively promoting progressive deterioration of liver, heart, kidney, pancreas and brain function [10,11]. These observations underscore the central role of mitochondrial dysfunction in metabolic disease progression and highlight preservation of mitochondrial function as an important therapeutic objective.

### 1.3. Why a Systems-Level Perspective Is Needed

Despite substantial advances in mitochondrial biology, obesity and T2D are increasingly recognized as complex disorders involving highly interconnected alterations in energy metabolism, redox regulation, inflammatory signaling, proteostasis, and tissue remodeling. These observations highlight the need for systems-level approaches capable of integrating coordinated biological responses rather than isolated molecular pathways [12,13]. Advances in quantitative proteomics now provide unprecedented opportunities to investigate these interactions at the network level, establishing the rationale for the conceptual framework proposed in this review.

### 1.4. Translational Potential of Curcumin

Curcumin has attracted considerable interest because experimental studies consistently demonstrate beneficial effects on mitochondrial function, oxidative stress, inflammation, and metabolic regulation across multiple organs [14,15]. Increasing evidence suggests that these pleiotropic actions extend beyond individual signaling pathways and may reflect coordinated modulation of interconnected mitochondrial biological processes [16,17]. Accordingly, curcumin is discussed throughout this review as a representative example of systems-level mitochondrial network modulation rather than as the central focus of the proposed conceptual framework.

## 2. Introduction

Despite major advances in mitochondrial biology and metabolic disease research, the mechanistic understanding of obesity and T2D remains largely based on reductionist models that emphasize individual molecular pathways or isolated signaling mechanisms. Although this approach has generated substantial mechanistic insight, it provides limited understanding of how coordinated mitochondrial dysfunction emerges across multiple metabolically active organs during chronic metabolic stress. Increasing evidence indicates that metabolic overload, oxidative stress, inflammation, tissue remodeling, and progressive organ dysfunction do not occur as independent pathological processes but rather represent highly interconnected biological responses that evolve through coordinated regulatory mechanisms. These observations highlight the need for integrative conceptual frameworks capable of explaining mitochondrial dysfunction at the systems level rather than through isolated molecular events.

Advances in quantitative mitochondrial proteomics have fundamentally expanded our understanding of metabolic disease by revealing coordinated patterns of mitochondrial remodeling that extend beyond individual molecular alterations. Comparative proteomic analyses performed across multiple organs and experimental models consistently demonstrate that mitochondrial proteins involved in OXPHOS, FAO, the tricarboxylic acid (TCA) cycle, redox homeostasis, proteostasis, and adaptive stress responses undergo remarkably conserved remodeling during obesity, T2D, and chronic nutrient excess [13,18]. Rather than representing isolated molecular events, these recurrent proteomic signatures suggest the existence of coordinated functional modules that collectively govern mitochondrial adaptation to metabolic stress.

Although comparative proteomics has consistently revealed conserved patterns of mitochondrial remodeling across metabolically active tissues, the biological significance of this functional convergence remains incompletely understood. Current mechanistic models primarily interpret these observations through individual signaling pathways or discrete molecular targets, providing limited insight into how conserved mitochondrial functional modules interact as integrated biological systems. Consequently, the central challenge is no longer the identification of additional mitochondrial proteins, but rather the development of conceptual frameworks capable of explaining how coordinated mitochondrial networks regulate metabolic adaptation and disease progression.

To address this conceptual gap, we propose the framework of Mitochondrial-Centered Biological Networks (MCBNs), in which conserved mitochondrial functional modules are viewed as dynamically interconnected biological networks rather than independent molecular pathways [12,19]. Building evidence from comparative mitochondrial proteomics, systems biology, and network medicine, this framework recognizes that mitochondrial adaptation emerges from coordinated interactions among core functional modules, including energy metabolism, FAO, TCA cycle, redox homeostasis, proteostasis, and adaptive stress responses. Within this systems-level organization, the biological behavior of the mitochondrial network cannot be fully understood by examining individual pathways in isolation but instead arises from the dynamic integration of these conserved functional modules.

Within the MCBN framework, mitochondrial homeostasis is conceptualized as an emergent systems-level property arising from the dynamic coordination of interconnected mitochondrial functional modules rather than from preservation of any single mitochondrial process. Building on this perspective, we introduce the Mitochondrial Homeostasis Hypothesis, which proposes that tissue adaptation during obesity and T2D depends on the maintenance or restoration of coordinated mitochondrial network integrity. Conversely, progressive disruption of these network interactions promotes the transition from adaptive remodeling toward mitochondrial dysfunction and metabolic disease progression.

Among the diverse interventions reported to influence mitochondrial function, curcumin represents one of the best-characterized bioactive compounds and serves in this review as a representative example of systems-level mitochondrial network modulation [15,20]. Experimental evidence indicates that curcumin modulates multiple aspects of mitochondrial biology, including bioenergetics, redox homeostasis, proteostasis, and adaptive stress responses [16,21].

**Figure 1:**
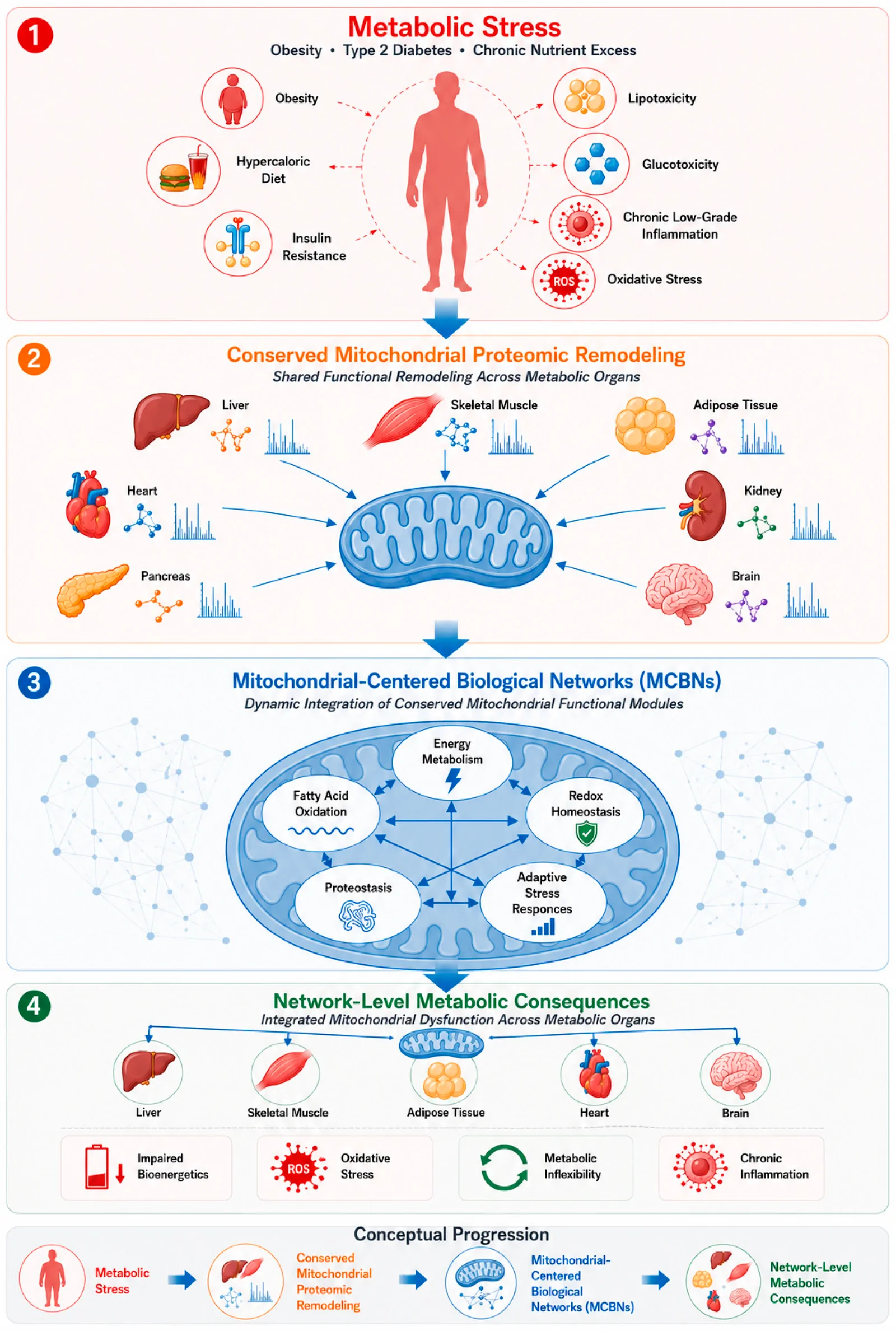
Conceptual Framework of Mitochondrial-Centered Biological Networks in Metabolic Disease. Chronic metabolic stress associated with obesity, T2D, and chronic nutrient excess induces conserved mitochondrial proteomic remodeling across multiple metabolically active organs. Comparative mitochondrial alterations converge into a limited number of conserved functional modules, including energy metabolism, FAO, TCA cycle activity, redox homeostasis, proteostasis, and adaptive stress responses, that collectively constitute MCBNs. This systems-level organization provides a unifying conceptual framework for understanding coordinated mitochondrial dysfunction across tissues and establishes the foundation for experimental evidence, network modulation, mitochondrial homeostasis hypothesis, and translational applications.

Accordingly, this narrative and conceptual review pursues five complementary objectives. First, we synthesize current proteomic evidence describing conserved mitochondrial remodeling during obesity and T2D. Second, we integrate these observations with contemporary advances in mitochondrial biology, redox signaling, systems biology, and network medicine to establish a unified systems-level perspective. Third, we introduce the conceptual framework of Mitochondrial-Centered Biological Networks (MCBNs) and distinguish it from existing models of mitochondrial dysfunction. Fourth, we examine curcumin as a representative example of mitochondrial network modulation within this conceptual framework. Finally, we discuss how emerging technologies, including quantitative proteomics, redox proteomics, spatial proteomics, single-cell proteomics, integrated multi-omics, and artificial intelligence, may facilitate experimental validation of the proposed framework and support future advances toward precision mitochondrial medicine. The overall conceptual framework developed throughout this review is summarized in Figure 1. Chronic metabolic stress induces conserved mitochondrial proteomic remodeling across multiple metabolically active organs. These recurrent proteomic alterations converge into interconnected MCBNs composed of conserved functional modules, providing the conceptual foundation for the experimental evidence, network modulation, mitochondrial homeostasis hypothesis, and translational perspectives presented in the subsequent sections.

### Literature Search Strategy

This article was designed as a narrative and conceptual review aimed at integrating evidence from comparative proteomics, mitochondrial biology, bioenergetics, redox signaling, systems biology, and metabolic disease research to develop the proposed framework of MCBNs and the Mitochondrial Homeostasis Hypothesis. Rather than following the methodology of a systematic review or meta-analysis, this review emphasizes the critical integration of multidisciplinary evidence to identify recurrent biological principles underlying mitochondrial adaptation during metabolic disease.

The literature search was performed using the PubMed, Web of Science, and Scopus databases. Searches were conducted from January 2024 through February, although seminal publications that established fundamental concepts in mitochondrial biology, redox regulation, proteomics, systems biology, and network medicine were also included regardless of publication date.

The principal search terms included combinations of “obesity,” “type 2 diabetes,” “mitochondria,” “mitochondrial dysfunction,” “proteomics,” “quantitative proteomics,” “oxidative phosphorylation,” “fatty acid oxidation,” “tricarboxylic acid cycle,” “redox signaling,” “oxidative stress,” “mitochondrial proteostasis,” “mitophagy,” “mitonuclear communication,” “mitohormesis,” “curcumin,” “systems biology,” “network medicine,” “spatial proteomics,” “single-cell proteomics,” and “multi-omics.”

Priority was given to original research articles, high-quality review articles, and recent peer-reviewed studies employing quantitative proteomics, mitochondrial functional analyses, bioinformatics, and systems biology approaches. Seminal publications were included when necessary to provide historical context for concepts that remain fundamental to contemporary mitochondrial biology, including mitochondrial quality control, mitonuclear communication, redox signaling, and network medicine.

Particular emphasis was placed on studies describing proteomic remodeling associated with obesity, T2D, chronic nutrient excess, and curcumin treatment. Whenever possible, proteomic observations were interpreted alongside complementary biochemical, physiological, molecular, and bioenergetic evidence to facilitate a systems-level interpretation of mitochondrial adaptation across tissues and experimental models.

Several proteomic investigations performed by our research group are discussed because they examined multiple metabolically relevant organs and experimental models using comparable proteomic and bioinformatic methodologies. This consistency provides a valuable opportunity to evaluate the reproducibility of mitochondrial proteomic remodeling across tissues while maintaining a balanced perspective through integration with independent studies from the broader literature.

Because the MCBN framework and the Mitochondrial Homeostasis Hypothesis are presented as conceptual models, the evidence discussed throughout this review should be interpreted as an integrative synthesis of current knowledge rather than as definitive experimental validation. Future advances in quantitative proteomics, redox proteomics, spatial proteomics, single-cell proteomics, integrated multi-omics, and artificial intelligence will be essential to experimentally evaluate, refine, and extend the proposed framework.

## 3. Mitochondrial-Centered Biological Networks in Metabolic Disease

### 3.1. From Mitochondrial Dysfunction to Network Biology

Mitochondrial dysfunction has long been recognized as a characteristic feature of obesity, T2D, and hypercaloric diet-induced metabolic dysfunction [3,9]. Traditionally, mitochondrial abnormalities have been described in terms of impaired oxidative phosphorylation, excessive reactive oxygen species (ROS) production, altered substrate utilization, and defective ATP generation. While these alterations remain central to disease pathophysiology, growing evidence indicates that they represent only part of a broader network-level adaptive response involving multiple interconnected regulatory systems.

Contemporary systems biology recognizes that complex diseases emerge from perturbations affecting biological networks rather than isolated molecular pathways [12,19]. Within this framework, mitochondria occupy a uniquely strategic position because they coordinate energy production, nutrient sensing, redox regulation, protein quality control, and cellular adaptation. Consequently, disturbances affecting mitochondrial function can propagate across multiple biological processes and contribute to widespread metabolic dysfunction.

This perspective suggests that obesity and T2D should be interpreted not only as disorders of glucose and lipid metabolism but also as diseases characterized by progressive destabilization of mitochondrial-centered regulatory networks. The diverse manifestations observed in metabolically stressed tissues may therefore represent organ-specific responses to common network-level disturbances.

### 3.2. Defining Mitochondrial-Centered Biological Networks

Accumulating evidence derived from mitochondrial biology, proteomics, bioenergetics, and systems medicine is consistent with the existence of highly interconnected biological modules centered on mitochondrial function [7,8]. Based on this evidence, we propose the concept of Mitochondrial-Centered Biological Networks (MCBNs).

MCBNs comprise interconnected molecular and functional systems that collectively regulate mitochondrial adaptation, cellular resilience and tissue homeostasis. Within these networks, mitochondria function simultaneously as bioenergetic hubs responsible for ATP production, metabolic sensors that respond to nutrient availability, redox regulators coordinating ROS signaling and antioxidant defenses, proteostasis regulators maintaining protein quality control, and integrative platforms that orchestrate adaptive responses to physiological and pathological stress.

Rather than operating independently, these functions interact continuously through dynamic feedback mechanisms. Consequently, perturbation of one network module frequently influences the behavior of others, generating system-wide responses that extend far beyond individual molecular pathways. Within this conceptual framework, mitochondrial homeostasis emerges as a systems-level property resulting from the coordinated regulation of multiple biological processes rather than from maintenance of any single mitochondrial function.

### 3.3. What Does the MCBN Framework Add to Existing Concepts in Mitochondrial Biology?

Over the past two decades, several concepts have substantially advanced our understanding of mitochondrial biology, including mitochondrial quality control (MQC), mitophagy, mitonuclear communication, mitohormesis, redox signaling, systems biology, and network medicine. Rather than replacing these well-established paradigms, the proposed MCBN framework integrates them into a unified systems-level organizational model that facilitates interpretation of the conserved mitochondrial proteomic remodeling observed across metabolically active tissues. As summarized in Table 1, the MCBN framework builds on these established concepts while extending their integration toward a proteomics-driven, network-oriented interpretation of mitochondrial adaptation during metabolic disease.

MQC encompasses the molecular processes that preserve mitochondrial integrity through mitochondrial biogenesis, fusion and fission dynamics, mitophagy, mitochondrial protein quality control, and the mitochondrial unfolded protein response (UPR^mt^) [22,23,24]. Within this framework, mitophagy represents a specialized quality-control mechanism responsible for the selective elimination of damaged mitochondria [22,25]. Together, these processes maintain organelle integrity and functionality by coordinating mitochondrial renewal and protein homeostasis. The proposed MCBN framework fully incorporates these mechanisms but extends beyond organelle maintenance by integrating MQC with coordinated remodeling of bioenergetics, substrate metabolism, redox regulation, proteostasis, adaptive signaling, and tissue-level responses, thereby placing MQC within a broader systems-level context (Table 1).

Mitochondrial adaptation also depends on signaling mechanisms that coordinate cellular responses to metabolic and environmental stress. Mitonuclear communication regulates the bidirectional exchange of information between mitochondria and the nucleus, thereby controlling mitochondrial biogenesis, oxidative metabolism, and stress-responsive transcriptional programs [23,26]. Complementing this regulatory axis, the concept of mitohormesis recognizes that moderate increases in mitochondrial ROS activate adaptive responses that enhance metabolic flexibility and cellular resilience.

**Table 1:** Comparison of the MCBN framework with established concepts in mitochondrial biology.

Concept	Primary Focus	Biological Level	Major Mechanisms	Main Limitation	Integration Within the MCBN Framework	Representative References
Mitochondrial dysfunction	Functional impairment of mitochondria during disease	Organelle	Impaired oxidative phosphorylation, ATP depletion, excessive ROS production, metabolic dysfunction	Primarily descriptive; does not explain how multiple adaptive pathways are coordinated	MCBNs interpret mitochondrial dysfunction as the progressive destabilization of interconnected biological networks rather than isolated organelle defects	Nunnari & Suomalainen, 2012 [7]; Zong et al., 2024 [27]
MQC	Preservation of mitochondrial integrity	Organelle	Mitochondrial biogenesis, fusion/fission dynamics, mitophagy, UPR^mt^, protein quality control	Focuses mainly on maintenance of mitochondrial integrity	MCBNs incorporate MQC as one functional module integrated with metabolism, redox regulation, proteostasis and signaling	Pickles et al., 2018 [22]; Ng et al., 2021 [24]
Mitophagy	Selective removal of damaged mitochondria	Organelle	PINK1/Parkin pathway, receptor-mediated mitophagy, lysosomal degradation	Addresses one quality-control mechanism but not systems-level adaptation	MCBNs consider mitophagy as one component of a broader adaptive mitochondrial network	Pickles et al., 2018 [22]; Onishi et al., 2021 [25]
Mitonuclear communication	Bidirectional signaling between mitochondria and nucleus	Organelle–Nucleus	Retrograde signaling, mitochondrial unfolded protein response, transcriptional regulation	Primarily focuses on intracellular communication	MCBNs extend this concept by integrating mitochondrial communication with metabolic, inflammatory and extracellular adaptive responses	Quirós et al., 2016 [23]; Mottis et al., 2019 [26]
Mitohormesis	Adaptive responses induced by mild mitochondrial stress	Cellular	ROS-mediated signaling, adaptive stress responses, metabolic adaptation	Mainly emphasizes redox signaling	MCBNs integrate hormetic responses with bioenergetics, lipid metabolism, proteostasis and tissue remodeling	Yun & Finkel, 2014 [28]; Merry & Ristow, 2016 [29]
Redox signaling	ROS as physiological signaling molecules	Molecular–Cellular	Thiol oxidation, antioxidant systems, Nrf2 activation, thioredoxin and glutathione systems	Primarily centered on oxidative signaling	MCBNs include redox signaling as one interconnected module among multiple mitochondrial adaptive systems	Sies & Jones, 2020 [30]; Forman & Zhang, 2021 [31]
Systems biology	Global organization of biological systems	Cellular–Organismal	Multi-scale integration of biological pathways	Does not specifically focus on mitochondrial organization	MCBNs apply systems biology principles specifically to mitochondrial-centered regulation during metabolic disease	Kitano, 2002 [32]; Loscalzo & Barabasi, 2011 [19]
Network medicine	Disease as perturbation of biological networks	Organismal	Network topology, disease modules, interactome analysis	Disease-oriented but not mitochondria-centered	MCBNs represent a mitochondrial-focused application of network medicine to obesity and type 2 diabetes	Barabási et al., 2011 [12]; Loscalzo & Barabasi, 2011 [19]
Mitochondrial-Centered Biological Networks (MCBNs) (proposed framework)	Integrated regulation of mitochondrial adaptation during metabolic stress	Multi-scale (molecule → organelle → cell → tissue → organ)	Coordinated remodeling of oxidative phosphorylation, fatty acid oxidation, TCA cycle, redox regulation, proteostasis, adaptive signaling, inflammation and tissue remodeling	Requires experimental validation in preclinical and human studies	Provides an integrative conceptual framework linking proteomic remodeling with mitochondrial homeostasis and systems-level adaptation in obesity and type 2 diabetes	Present review; supported by proteomic evidence discussed herein

Abbreviations: MQC, mitochondrial quality control; MCBNs, mitochondrial-centered biological networks; ROS, reactive oxygen species; TCA, tricarboxylic acid cycle; UPR^mt^, mitochondrial unfolded protein response.

Within the proposed MCBN framework, these mechanisms are interpreted as interconnected adaptive signaling modules that operate in concert with bioenergetic, metabolic, redox, and proteostatic networks rather than as isolated regulatory pathways (Table 1). At a broader organizational level, systems biology and network medicine have fundamentally changed the interpretation of complex diseases by proposing that pathological phenotypes emerge from disturbances affecting interconnected biological networks rather than isolated molecular pathways [12,19]. The MCBN framework adopts these principles but applies them specifically to mitochondrial adaptation during obesity and T2D. Its novelty does not reside in identifying new mitochondrial mechanisms, but in integrating comparative proteomic remodeling, mitochondrial bioenergetics, substrate utilization, redox regulation, proteostasis, adaptive signaling, inflammatory responses, and tissue remodeling into a unified mitochondrial-centered organizational model. This perspective is further supported by growing evidence demonstrating that mitochondrial dysfunction influences immune regulation, interorgan communication, and disease progression through highly interconnected biological networks [27,33].

A defining feature of the MCBN framework is its close relationship with advances in comparative mitochondrial proteomics. Unlike reductionist approaches centered on individual signaling pathways, quantitative proteomic analyses consistently reveal coordinated remodeling of proteins involved in OXPHOS, FAO, TCA cycle activity, redox regulation, proteostasis, and adaptive stress responses across multiple tissues and experimental models [34]. These recurrent observations support the interpretation that mitochondrial dysfunction during metabolic disease reflects coordinated network remodeling rather than isolated molecular alterations [35]. Accordingly, MCBNs provide a conceptual framework for organizing these proteomic observations into a biologically coherent and experimentally testable model of mitochondrial adaptation.

Accordingly, the MCBN framework should be regarded as a working conceptual model rather than an established biological theory. By integrating current knowledge from comparative proteomics, mitochondrial biology, bioenergetics, redox signaling, and systems medicine, it provides an organizational framework for interpreting coordinated mitochondrial remodeling and generating experimentally testable hypotheses regarding metabolic adaptation and disease progression. Future advances in quantitative proteomics, redox proteomics, spatial proteomics, single-cell proteomics, integrated multi-omics, and artificial intelligence will be essential to evaluate, refine, and validate this proposed framework in both experimental models and human metabolic disease [36,37].

### 3.4. Core Functional Modules of MCBNs

Although mitochondrial biology encompasses numerous interconnected pathways, comparative proteomic studies consistently identify a limited number of functional modules that undergo coordinated remodeling across metabolically active tissues during obesity, T2D, and chronic nutrient excess. Within the proposed MCBN framework, these recurrently affected modules represent the core organizational units through which mitochondrial adaptation is interpreted [34].

#### 3.4.1. Bioenergetic Networks

Bioenergetic networks comprise interconnected pathways responsible for OXPHOS, electron transport, ATP synthesis, and mitochondrial energy conversion. These processes determine cellular energetic capacity and metabolic flexibility, allowing tissues to adapt to fluctuations in nutrient availability and energetic demand. During obesity, T2D, and chronic nutrient excess, coordinated impairment of these pathways compromises ATP production, increases oxidative stress, and contributes to metabolic dysfunction [3,8]. Within the MCBN framework, bioenergetic remodeling is interpreted as a systems-level alteration affecting an integrated functional module rather than isolated respiratory-chain components.

#### 3.4.2. Metabolic Networks

Metabolic networks comprise the interconnected pathways that regulate substrate utilization, TCA cycle activity, FAO, and the integration of carbohydrate and lipid metabolism. The coordinated regulation of these processes is essential for maintaining metabolic flexibility and enabling cells to adapt efficiently to changes in nutrient availability and energetic demand [35]. During obesity, T2D, and chronic nutrient excess, disruption of these pathways contributes to impaired lipid utilization, accumulation of lipotoxic intermediates, altered mitochondrial substrate selection, and progressive metabolic dysfunction [10]. Accordingly, metabolic networks provide a functional interface through which mitochondrial bioenergetics is coordinated with substrate utilization and metabolic flexibility within the MCBN framework.

#### 3.4.3. Redox Regulatory Networks

Redox regulatory networks comprise the interconnected molecular systems that maintain the balance between ROS production and antioxidant defenses, thereby preserving redox homeostasis during physiological and pathological conditions. Mitochondria are simultaneously major sources and targets of ROS, placing redox regulation at the center of cellular adaptation to metabolic stress. Under physiological conditions, ROS function as essential signaling molecules that regulate mitochondrial biogenesis, metabolic adaptation, and stress-responsive pathways [30]. However, persistent nutrient overload and mitochondrial dysfunction promote excessive ROS production, leading to oxidative damage, disruption of redox homeostasis, and progressive destabilization of interconnected biological networks [38,39]. Within the MCBNs, redox regulation is therefore viewed as an integrated adaptive module that coordinates mitochondrial function with cellular stress responses and metabolic homeostasis.

#### 3.4.4. Proteostasis Networks

Mitochondrial proteostasis comprises the interconnected processes responsible for maintaining protein homeostasis within mitochondria, including protein import, folding, assembly, quality control, and selective degradation. These mechanisms, coordinated by molecular chaperones, ATP-dependent proteases, the UPR^mt^, and mitophagy, preserve mitochondrial integrity by preventing the accumulation of damaged or misfolded proteins and ensuring proper mitochondrial function [40,41]. During obesity, T2D, and chronic nutrient excess, impairment of these proteostatic mechanisms contributes to progressive mitochondrial dysfunction, diminished adaptive capacity, and increased susceptibility to metabolic stress [22,23,35]. Accordingly, mitochondrial proteostasis contributes to network stability by preserving protein quality, organelle integrity, and adaptive capacity during metabolic stress.

#### 3.4.5. Adaptive Signaling Networks

Adaptive signaling networks integrate information regarding nutrient availability, cellular energy status, oxidative stress, and environmental stimuli to coordinate mitochondrial adaptation and maintain cellular homeostasis [35]. These interconnected signaling pathways regulate mitochondrial biogenesis, metabolic flexibility, stress responses, and tissue resilience in response to changing physiological and pathological conditions. Among the principal regulatory pathways, AMP-activated protein kinase (AMPK), sirtuin 1 (SIRT1), peroxisome proliferator-activated receptor gamma coactivator-1 alpha (PGC-1α), peroxisome proliferator-activated receptors (PPARα and PPARγ), and nuclear factor erythroid 2-related factor 2 (Nrf2) promote mitochondrial adaptation by coordinating energy sensing, oxidative metabolism, antioxidant defense, and mitochondrial biogenesis [42]. Conversely, sustained activation of stress and inflammation-associated pathways, including nuclear factor-kappa B (NF-κB), c-Jun N-terminal kinase (JNK), and transforming growth factor-beta (TGF-β), contributes to impaired mitochondrial adaptation, chronic inflammation, fibrotic remodeling, and progressive metabolic dysfunction [43,44]. Adaptive signaling networks therefore provide the regulatory interface that dynamically coordinates mitochondrial bioenergetics, metabolism, redox balance, and proteostasis within the MCBN framework [36].

### 3.5. Network Destabilization During Metabolic Disease Progression

Under physiological conditions, the coordinated interaction of bioenergetic, metabolic, redox, proteostatic, and adaptive signaling networks preserves mitochondrial homeostasis, metabolic flexibility, and tissue resilience. Within the proposed MCBN framework, these interconnected modules enable mitochondria to dynamically adapt to changes in nutrient availability while maintaining cellular function under fluctuating metabolic demands [35].

Chronic caloric excess progressively disrupts this coordinated regulation. Sustained nutrient overload increases mitochondrial electron transport activity, promotes excessive ROS production, alters substrate utilization, and imposes persistent metabolic stress [3,45]. Although compensatory mechanisms, including antioxidant defenses, mitochondrial biogenesis, and protein quality-control pathways, initially preserve mitochondrial function, prolonged metabolic overload may eventually exceed the adaptive capacity of these interconnected networks, leading to progressive loss of mitochondrial resilience [46].

Accordingly, metabolic disease progression is interpreted within the MCBN framework as the gradual destabilization of interconnected functional networks rather than the consequence of isolated molecular defects [34,46]. Progressive impairment of bioenergetic, metabolic, redox, proteostatic, and adaptive signaling modules collectively reduces metabolic flexibility, amplifies oxidative stress and inflammation, and increases tissue susceptibility to dysfunction. This systems-level interpretation provides the conceptual bridge between mitochondrial proteomic remodeling and the development of obesity, T2D, and their associated complications [37].

### 3.6. Organ-Specific Manifestations of a Common Network Disturbance

The systems-level nature of MCBNs becomes particularly evident when examining the organ-specific manifestations of metabolic disease. Thus, the clinical diversity of metabolic disease may reflect not fundamentally different mitochondrial mechanisms, but rather tissue-specific manifestations of a shared MCBN architecture operating under distinct physiological and metabolic contexts [35].

The specific consequences of mitochondrial network disruption differ according to the physiological specialization and metabolic demands of each organ. In the liver, impaired mitochondrial adaptation contributes to steatosis, altered glucose production, and metabolic inflexibility; in the heart, it promotes energetic dysfunction, lipotoxicity, and diabetic cardiomyopathy; in the kidney, persistent mitochondrial stress favors oxidative damage, inflammation, and fibrotic remodeling; whereas pancreatic β-cells exhibit impaired glucose sensing and insulin secretion, and the brain develops oxidative stress, neuroinflammation, and cognitive decline [8,9,11,45]. Despite these distinct phenotypic manifestations, comparative proteomic analyses performed across multiple metabolically active tissues have repeatedly demonstrated convergent remodeling of the same functional modules, including OXPHOS, FAO, redox regulation, proteostasis, and adaptive signaling, supporting the concept that diverse clinical outcomes arise from a shared MCBN architecture operating under distinct physiological and metabolic contexts [34,35,37].

### 3.7. Mitochondrial Homeostasis as an Emergent Property of MCBNs

Within the MCBN framework, mitochondrial homeostasis is conceptualized as an emergent systems-level property arising from the coordinated interaction of bioenergetic, metabolic, redox, proteostatic, and adaptive signaling networks rather than from the integrity of any individual pathway. When this network organization is preserved, tissues maintain efficient energy utilization, metabolic flexibility, controlled redox signaling, and adaptive resilience. Conversely, progressive loss of coordination among these functional modules may impair mitochondrial adaptation, amplify oxidative stress and inflammation, promote fibrotic remodeling, and ultimately contribute to organ dysfunction [34,35].

This systems-level interpretation provides the conceptual foundation for the Mitochondrial Homeostasis Hypothesis proposed in this review. According to this hypothesis, metabolic disease progression reflects the gradual destabilization of interconnected MCBNs, whereas interventions capable of preserving or restoring network integrity may enhance adaptive capacity and tissue resilience. The hypothesis should therefore be regarded as a testable conceptual model whose experimental evaluation will require integration of comparative proteomics with complementary multi-omics, functional, and translational approaches.

## 4. Proteomic Evidence Supporting Mitochondrial-Centered Biological Networks

### 4.1. Proteomics Reveals Coordinated Network Remodeling During Metabolic Disease

The application of proteomic technologies to obesity and T2D research has fundamentally transformed our understanding of metabolic disease progression. Unlike traditional biochemical approaches focused on a limited number of molecular targets, mass spectrometry-based proteomics enables the simultaneous characterization of hundreds to thousands of proteins, providing a comprehensive view of the molecular adaptations that occur during chronic metabolic stress [13,18,47]. Beyond identifying differentially expressed proteins, comparative proteomic analyses reveal coordinated changes across interconnected biological processes, allowing mitochondrial remodeling to be interpreted at the level of functional networks rather than isolated molecular events [34,48,49].

One of the most consistent observations emerging from independent proteomic studies is that obesity, T2D, and chronic nutrient excess induce reproducible remodeling of a limited number of conserved mitochondrial functional modules. Across diverse experimental models, tissues, and analytical platforms, recurrent alterations involve OXPHOS, TCA cycle, FAO, redox regulation, mitochondrial proteostasis, and adaptive stress responses [37]. Rather than representing unrelated molecular abnormalities, the convergence of these findings supports the interpretation that metabolic disease is characterized by coordinated reorganization of interconnected mitochondrial processes. The following sections examine the proteomic evidence supporting each of these functional modules and illustrate how their collective remodeling provides the experimental foundation for the proposed MCBN framework and the Mitochondrial Homeostasis Hypothesis.

### 4.2. Foundational Proteomic Evidence of Coordinated Mitochondrial Remodeling

Proteomic analyses of liver tissue from diabetic obese db/db mice provided some of the first evidence that metabolic disease is associated with coordinated mitochondrial remodeling rather than isolated molecular alterations [50,51]. Although these studies were originally designed to identify proteins associated with diabetes progression, subsequent bioinformatic reanalysis revealed significant enrichment of mitochondrial pathways involved in oxidative metabolism, redox regulation, and cellular adaptation [47]. These findings retrospectively anticipated the concept that chronic metabolic stress promotes concerted reorganization of interconnected mitochondrial functional modules.

Among the proteins consistently altered were pyruvate carboxylase (PCX), succinate dehydrogenase (SDHA), carbamoyl phosphate synthase 1 (CPS1), and ornithine aminotransferase (OAT), representing key components of mitochondrial carbon metabolism, respiratory function, nitrogen metabolism, and amino acid homeostasis [45]. Rather than emphasizing the biological significance of individual proteins, these datasets revealed coordinated perturbations affecting multiple mitochondrial processes simultaneously. These pioneering observations provided the first experimental evidence that metabolic disease is accompanied by integrated remodeling of conserved mitochondrial functional modules, a concept that ultimately forms the biological basis of the MCBN framework.

### 4.3. Oxidative Phosphorylation Networks as Recurrent Proteomic Targets

Among the mitochondrial functional modules identified across proteomic datasets, OXPHOS is one of the most consistently remodeled during obesity, T2D, and chronic nutrient excess. Because OXPHOS integrates electron transport, ATP synthesis, oxygen consumption, and redox balance, coordinated alterations within this module have broad consequences for cellular bioenergetics, metabolic flexibility, and mitochondrial adaptation [35]. Independent studies performed in liver, heart, skeletal muscle, kidney, and pancreas consistently demonstrate recurrent remodeling of proteins associated with respiratory chain function, indicating that disturbances of oxidative metabolism represent a conserved feature of metabolic disease rather than tissue-specific abnormalities.

Representative proteins recurrently identified across these studies include the complex I subunits NDUFS1 and NDUFV1, the complex II component SDHA, ubiquinol-cytochrome c reductase core protein 1 (UQCRC1), cytochrome c oxidase subunit COX5A, and the ATP synthase subunits ATP5A1 and ATP5B [47]. Functional enrichment analyses further reinforce these observations by consistently identifying oxidative phosphorylation, cellular respiration, ATP synthesis, and mitochondrial energy metabolism among the biological processes most significantly affected during obesity and T2D progression [8,25]. Together, these findings indicate that metabolic stress remodels the bioenergetic machinery as an integrated functional system rather than through isolated alterations of individual respiratory proteins.

From the perspective of the proposed MCBN framework, remodeling of the OXPHOS module should be interpreted as one component of a broader adaptive response involving coordinated changes in metabolic, redox, proteostatic, and signaling networks. Thus, alterations in OXPHOS are not viewed as isolated defects but as part of an integrated reorganization of mitochondrial function during chronic metabolic stress [37].

### 4.4. Fatty Acid Oxidation Networks

FAO constitutes another conserved mitochondrial functional module that undergoes reproducible remodeling during obesity, type 2 diabetes, and chronic nutrient excess. As the principal pathway responsible for mitochondrial lipid utilization, FAO plays a central role in maintaining metabolic flexibility by matching substrate oxidation to nutrient availability and energetic demand [45]. Persistent nutrient overload, however, progressively disrupts this adaptive capacity, promoting lipid accumulation, metabolic inflexibility, and mitochondrial dysfunction [10,35]. Comparative proteomic studies performed across multiple metabolically active tissues consistently identify alterations in proteins involved in mitochondrial fatty acid transport and β-oxidation, indicating that FAO remodeling represents a common feature of metabolic disease rather than an organ-specific phenomenon [47].

Representative proteins identified across independent datasets include carnitine palmitoyltransferases CPT1 and CPT2, together with key β-oxidation enzymes such as very-long-chain acyl-CoA dehydrogenase (VLCAD), medium-chain acyl-CoA dehydrogenase (MCAD), hydroxyacyl-CoA dehydrogenase alpha subunit (HADHA), and acetyl-CoA acyltransferase 2 (ACAA2) [47]. Although several of these proteins were initially described in cardiac proteomic studies evaluating curcumin, similar alterations have subsequently been reported in liver, skeletal muscle, and other metabolically active tissues. Functional enrichment analyses consistently identify FAO, mitochondrial β-oxidation, lipid metabolism, and substrate utilization, among the pathways most affected during metabolic disease progression, reinforcing the interpretation that FAO remodeling reflects coordinated adaptation of an entire metabolic module rather than isolated enzymatic changes.

Within this conceptual framework, remodeling of the FAO module extends beyond reduced ATP production. Alterations in this network influence metabolic flexibility, redox balance, bioenergetic efficiency, and mitochondrial signaling, thereby affecting the stability of multiple interconnected functional modules. The reproducibility of these proteomic observations across diverse biological contexts further supports the concept that mitochondrial adaptation during metabolic disease reflects integrated network reorganization rather than isolated defects in lipid metabolism.

### 4.5. Redox Regulatory Networks as Recurrent Proteomic Targets

Redox regulation represents a central integrative module within mitochondrial biology because it links energy metabolism, substrate utilization, stress signaling, and adaptive responses. Under physiological conditions, ROS function as tightly regulated signaling molecules that coordinate mitochondrial biogenesis, metabolic adaptation, and cellular homeostasis. During obesity, T2D, and chronic nutrient excess, however, persistent oxidative stress disrupts this regulatory balance, contributing to progressive mitochondrial dysfunction and impaired cellular adaptation [46]. Comparative proteomic studies consistently identify redox regulation among the mitochondrial processes most extensively remodeled during metabolic disease progression.

Independent proteomic datasets recurrently identify alterations in proteins participating in complementary antioxidant systems, including the peroxiredoxins PRDX1 and PRDX3, thioredoxin (TXN) and thioredoxin reductase (TXNRD1), glutathione-dependent enzymes such as GPX1 and members of the glutathione S-transferase (GST) family, together with the mitochondrial antioxidant enzyme superoxide dismutase 2 (SOD2). In parallel, advances in redox proteomics have revealed extensive remodeling of redox-sensitive post-translational modifications (PTMs), expanding our understanding of how oxidative signals regulate mitochondrial adaptation beyond changes in protein abundance [47].

Functional enrichment analyses consistently identify oxidative stress responses, glutathione metabolism, oxidoreductase activity, ROS metabolism, and cellular detoxification among the biological processes most significantly altered during metabolic disease progression. According to the MCBN model, redox regulation functions as an organizational interface connecting mitochondrial bioenergetics, substrate metabolism, proteostasis, and adaptive signaling. Consequently, disruption of redox homeostasis is interpreted not simply as an increase in oxidative damage but as evidence of network-wide reorganization affecting multiple interconnected functional modules. The reproducibility of these proteomic observations across experimental models further supports the concept that maintenance of redox balance is an essential component of mitochondrial homeostasis during metabolic adaptation.

### 4.6. Mitochondrial Proteostasis as an Emerging Network Module

Mitochondrial proteostasis has emerged as another recurrent functional module consistently identified across comparative proteomic studies of obesity, T2D, and chronic nutrient excess. Beyond sustaining protein quality, proteostasis preserves the structural and functional integrity of the mitochondrial proteome, thereby supporting respiratory activity, metabolic flexibility, stress adaptation, and organelle maintenance [47]. Consequently, disturbances in proteostatic mechanisms have implications that extend well beyond protein turnover, influencing the stability of multiple interconnected mitochondrial processes during metabolic stress [22,23].

Maintenance of mitochondrial proteostasis depends on the coordinated activity of molecular chaperones, ATP-proteases, protein import machinery, the UPR^mt^, and mitophagy pathways [52]. Comparative proteomic studies recurrently identify alterations in key components of these quality-control systems, including the molecular chaperones HSPD1 and HSPA9, the mitochondrial proteases CLPP and LONP1, together with additional proteins involved in mitochondrial protein turnover and organelle maintenance [35]. Functional enrichment analyses similarly identify protein folding, mitochondrial protein import, proteolysis, unfolded protein response, and organelle maintenance among the biological processes most consistently affected during metabolic disease progression.

Viewed through the MCBN framework, mitochondrial proteostasis functions as the structural maintenance system that preserves network integrity under conditions of metabolic stress. Progressive disruption of this module may compromise the coordinated operation of bioenergetic, metabolic, redox, and adaptive signaling networks by allowing the accumulation of damaged proteins and reducing mitochondrial resilience. Conversely, preservation of proteostatic capacity supports mitochondrial homeostasis by maintaining the functional organization required for coordinated adaptation across interconnected MCBNs.

### 4.7. Post-Translational Remodeling of Mitochondrial Networks: Carbonyl Stress and Protein Glycation

Proteomic remodeling during metabolic disease extends beyond changes in protein abundance to include PTMs that alter protein structure, stability, and function. Among these, carbonyl stress and advanced glycation reactions have emerged as important mechanisms linking chronic nutrient excess with mitochondrial dysfunction [53]. Consequently, mitochondrial adaptation should be interpreted as a combination of quantitative changes in protein expression and qualitative modifications that directly influence protein activity, molecular interactions, and network organization.

Proteomic studies demonstrated that fructose exposure and chronic metabolic overload promote glycation of proteins involved in OXPHOS, the TCA cycle, and mitochondrial substrate metabolism [54]. Rather than affecting isolated proteins, these modifications involve multiple interconnected pathways, indicating that PTMs remodel mitochondrial function at the level of coordinated biological networks. Consistent with this interpretation, recent advances in glycoproteomics and carbonyl proteomics have expanded our understanding of how metabolic stress reshapes the mitochondrial proteome beyond differential protein expression [55].

Within the MCBN framework, post-translational remodeling represents an additional regulatory dimension through which interconnected mitochondrial functional modules are dynamically modified during metabolic disease progression. Network remodeling therefore reflects both quantitative alterations in protein abundance and qualitative changes that influence enzymatic activity, molecular interactions, and structural integrity. Experimental studies showing that curcumin attenuates carbonyl stress and protein glycation [53] illustrate how representative interventions may preserve mitochondrial network organization, rather than supporting a mechanism unique to a single compound. Collectively, these findings broaden the biological scope of the MCBN framework by incorporating qualitative proteome remodeling as an integral component of mitochondrial homeostasis.

### 4.8. Functional Enrichment Analyses Reveal Convergent Biological Processes

Although differential protein expression identifies individual molecular alterations, the strongest evidence supporting the MCBN framework emerges from pathway enrichment analyses, which reveal reproducible convergence of mitochondrial functional modules across independent proteomic datasets [56,57].

Collective reanalysis of available proteomic datasets consistently demonstrates reduced enrichment of pathways associated with OXPHOS, ATP synthesis, FAO, TCA cycle, and mitochondrial organization, indicating a progressive decline in mitochondrial bioenergetic capacity and metabolic flexibility during obesity and T2D. In contrast, pathways involved in oxidative stress responses, inflammatory signaling, protein folding, extracellular matrix remodeling, and fibrosis are reproducibly enriched, reflecting activation of adaptive and pathological responses to chronic metabolic stress. Although the specific proteins contributing to these pathways may vary among tissues and experimental models, the biological processes identified remain remarkably consistent across independent studies.

This reproducible convergence of biological processes represents one of the strongest lines of evidence supporting the proposed MCBN framework. Unlike individual protein alterations, which may differ according to tissue type, disease stage, or analytical platform, pathway enrichment analyses consistently identify the same interconnected functional modules across independent proteomic studies [56,57]. This systems-level convergence indicates that metabolic disease is characterized by coordinated reorganization of conserved mitochondrial biological networks rather than by isolated molecular abnormalities. Accordingly, pathway enrichment analyses provide a direct experimental bridge between comparative proteomics and the Mitochondrial Homeostasis Hypothesis by demonstrating that recurrent patterns of mitochondrial remodeling emerge despite considerable biological heterogeneity. Representative recurrent proteins together with their associated enriched biological processes identified across independent proteomic datasets are summarized in Table 2.

### 4.9. Cross-Organ Proteomic Convergence

One of the most compelling observations emerging from comparative mitochondrial proteomics is the remarkable convergence of conserved functional modules across metabolically active organs. Although obesity, T2D, and chronic nutrient excess produce tissue-specific pathological manifestations, independent proteomic studies consistently identify remodeling of the same mitochondrial processes governing bioenergetics, substrate utilization, redox regulation, proteostasis, and adaptive responses. These findings indicate that metabolic disease disrupts a conserved biological organization that extends beyond the specialized physiology of individual organs [12].

This convergence is biologically plausible because all metabolically active tissues depend on a common repertoire of mitochondrial functions to sustain OXPHOS, substrate metabolism, redox homeostasis, proteostasis, and adaptive signaling [33]. Consequently, chronic metabolic stress perturbs these conserved processes regardless of the primary organ involved, whereas tissue-specific phenotypes largely reflect differences in physiological context rather than fundamentally distinct mechanisms of mitochondrial adaptation.

Comparative analyses across multiple organs further demonstrate that, despite differences in individual protein composition, pathway enrichment consistently identifies the same interconnected functional modules. This reproducible convergence provides biological support for interpreting mitochondrial remodeling as a coordinated adaptive response rather than a collection of tissue-specific molecular abnormalities. Collectively, these observations reinforce the concept that MCBNs represent conserved organizational units shared across metabolically active tissues.

**Figure 2:**
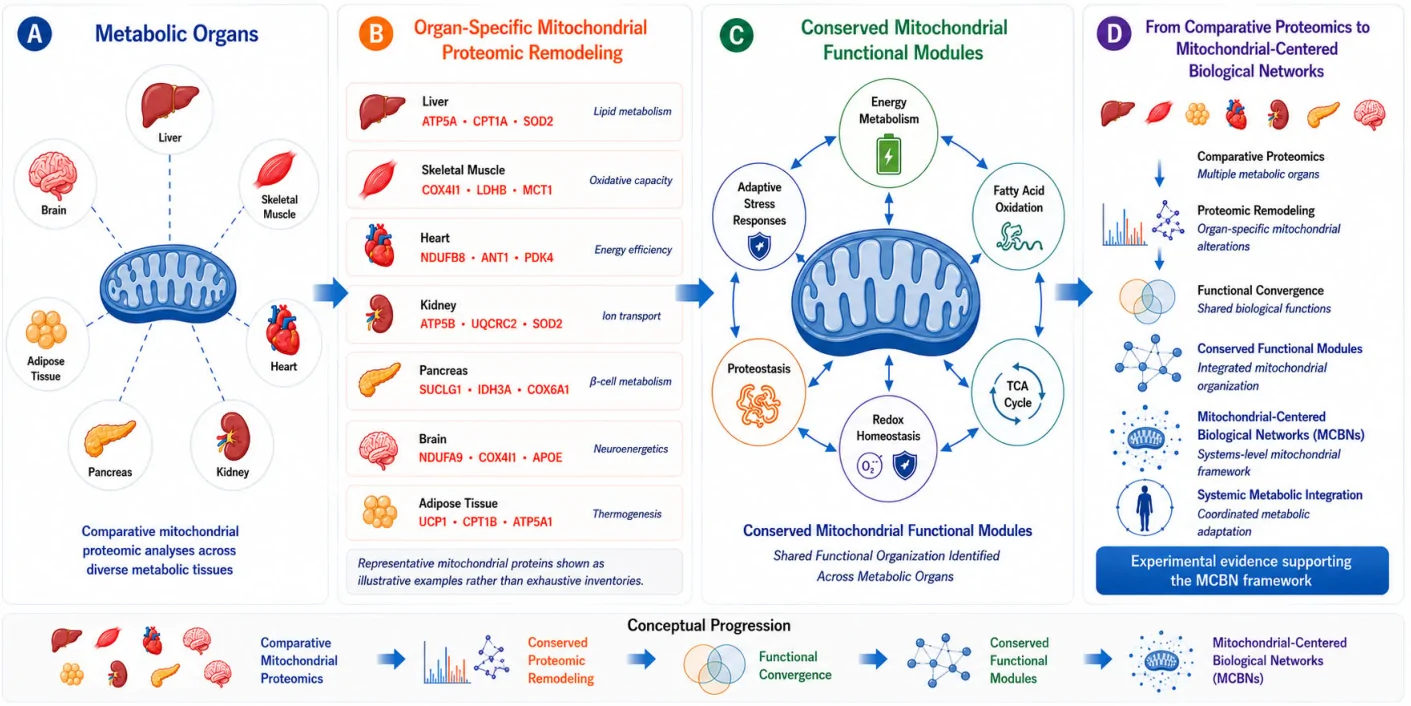
Proteomic Evidence Supporting Conserved Mitochondrial Functional Modules Across Metabolic Organs. Comparative mitochondrial proteomic studies performed in liver, skeletal muscle, heart, kidney, pancreas, brain, and adipose tissue consistently identify organ-specific mitochondrial protein remodeling, FAO, TCA cycle activity, redox homeostasis, proteostasis, and adaptive stress responses. (**A**) Comparative mitochondrial proteomic studies performed in liver, skeletal muscle, heart, kidney, pancreas, brain, and adipose tissue consistently identify organ-specific mitochondrial protein remodeling. (**B**) Despite tissue-specific proteomic signatures, these studies reveal highly conserved mitochondrial functional modules involved in FAO, TCA cycle activity, OXPHOS, redox homeostasis, proteostasis, mitochondrial dynamics, calcium homeostasis, mitochondrial DNA maintenance, mitophagy, and adaptive stress responses. (**C**) Integration of these conserved functional modules supports coordinated mitochondrial communication across metabolically active tissues, providing the conceptual basis for MCBNs. (**D**) The proposed MCBN framework illustrates how conserved mitochondrial functional modules coordinate tissue-specific adaptation while preserving systems-level mitochondrial homeostasis.

Accordingly, preservation of mitochondrial bioenergetics, metabolic flexibility, redox balance, and proteostatic capacity may represent common mechanisms that support tissue resilience during chronic metabolic stress. The cross-organ convergence revealed by comparative proteomics therefore provides the experimental rationale for considering mitochondrial homeostasis as an emergent property of MCBNs. Representative studies supporting this concept are summarized in Table 3 and Figure 2.

### 4.10. Proteomics as the Foundation of the Mitochondrial Homeostasis Hypothesis

Viewed as an integrated body of evidence, the comparative proteomic studies reviewed herein consistently demonstrate that metabolic disease is accompanied by reproducible remodeling of a limited number of conserved mitochondrial functional modules. Although early investigations focused primarily on identifying differentially expressed proteins, subsequent advances in quantitative proteomics, bioinformatics, and pathway enrichment analyses revealed recurring biological patterns that extend across tissues, experimental models, and analytical platforms [12].

Importantly, several representative interventions, including curcumin, have been shown to influence many of these conserved functional modules, including OXPHOS, FAO, redox regulation, proteostasis, and adaptive signaling [33]. These observations suggest that therapeutic benefit may arise from coordinated modulation of interconnected mitochondrial processes rather than selective regulation of individual molecular targets.

Collectively, the cumulative proteomic evidence supports the interpretation that mitochondrial homeostasis emerges from the coordinated behavior of interconnected functional networks rather than from the preservation of isolated mitochondrial pathways. Accordingly, progressive destabilization of these conserved modules provides a biologically plausible explanation for the metabolic, oxidative, inflammatory, and structural alterations that characterize obesity, T2D, and chronic nutrient excess.

Although the proposed Mitochondrial Homeostasis Hypothesis requires further experimental validation, the remarkable convergence of comparative proteomic evidence reviewed throughout this article provides a strong conceptual foundation for future investigations integrating quantitative proteomics, spatial and redox proteomics, single-cell technologies, functional analyses, and multi-omics approaches. More broadly, this systems-level perspective offers a framework for understanding mitochondrial adaptation and may facilitate the development of precision mitochondrial medicine for metabolic disease [63].

## 5. Functional and Signaling Consequences of MCBN Remodeling

### 5.1. From Proteomic Remodeling to Functional Adaptation

Comparative proteomic analyses provide a comprehensive molecular framework for understanding mitochondrial remodeling during obesity, T2D, and chronic exposure to hypercaloric diets. However, the biological significance of these molecular changes lies not in altered protein abundance itself, but in their functional consequences for mitochondrial performance, metabolic flexibility, stress adaptation, and tissue physiology. Consequently, interpretation of proteomic datasets requires integration with complementary bioenergetic, biochemical, and physiological evidence to determine how coordinated remodeling of mitochondrial functional modules translates into impaired cellular adaptation and disease progression.

An important conclusion emerging from the studies reviewed throughout this article is that functional impairment reflects progressive destabilization of interconnected mitochondrial functional modules rather than isolated alterations in individual proteins. Studies integrating comparative proteomics with measurements of mitochondrial respiration, oxidative stress, inflammation, fibrosis, and metabolic adaptation consistently demonstrate close agreement between molecular remodeling and functional decline. Collectively, these observations establish the experimental bridge linking proteomic remodeling to mitochondrial dysfunction and provide the functional rationale for the proposed MCBN framework.

### 5.2. Bioenergetic Consequences of OXPHOS Remodeling

Coordinated remodeling of the OXPHOS module has profound functional consequences because mitochondrial respiration underlies ATP production, metabolic flexibility, and cellular adaptation to energetic stress. Comparative proteomic studies consistently associate disruption of respiratory chain proteins with impaired mitochondrial respiration, reduced oxidative capacity, and diminished bioenergetic resilience during obesity, T2D, and chronic nutrient excess [33]. Rather than reflecting isolated defects in individual respiratory complexes, these findings indicate that bioenergetic dysfunction arises from destabilization of an integrated mitochondrial energy-producing network.

Reduced respiratory efficiency extends beyond diminished ATP synthesis. Progressive impairment of electron transport limits the capacity of mitochondria to respond to fluctuating energetic demands while simultaneously increasing electron leakage and oxidative stress. As bioenergetic performance declines, mitochondrial adaptability is progressively compromised, reducing metabolic flexibility and increasing tissue vulnerability to inflammatory and environmental challenges. These functional consequences help explain why disruption of the OXPHOS module influences multiple downstream biological processes rather than cellular energy production alone.

Interventions capable of preserving mitochondrial respiratory function are also associated with maintenance of OXPHOS-related proteomic organization and improved cellular bioenergetics [3,64]. Among these, curcumin represents a well-characterized experimental example that preserves mitochondrial respiration while maintaining expression of representative proteins within the OXPHOS module. These observations support the concept that functional recovery is more closely associated with stabilization of coordinated bioenergetic networks than with regulation of individual respiratory proteins.

Within the MCBN framework, preservation of mitochondrial bioenergetics represents a systems-level property that supports cellular adaptability, metabolic resilience, and maintenance of tissue function during chronic metabolic stress. Consequently, restoration of respiratory performance should be interpreted not simply as recovery of ATP production but as evidence of improved stability of interconnected mitochondrial biological networks.

### 5.3. Metabolic Consequences: Impaired Fatty Acid Oxidation and Loss of Metabolic Flexibility

Coordinated remodeling of the FAO module has important functional consequences because mitochondrial lipid utilization is essential for maintaining metabolic flexibility during changing nutritional and energetic conditions. Comparative proteomic studies consistently associate disruption of proteins involved in mitochondrial fatty acid transport and β-oxidation with reduced lipid utilization, impaired substrate switching, and progressive metabolic inflexibility during obesity, T2D, and chronic nutrient excess [33,61]. Rather than reflecting isolated defects in individual enzymes, these alterations indicate destabilization of an integrated metabolic network that limits the ability of mitochondria to adapt efficiently to fluctuations in energy demand.

Loss of metabolic flexibility has consequences that extend beyond impaired lipid oxidation. Reduced FAO promotes intracellular accumulation of lipid intermediates, increases lipotoxic stress, alters mitochondrial substrate selection, and progressively compromises insulin sensitivity. These metabolic disturbances further amplify oxidative stress and bioenergetic dysfunction, illustrating how disruption of a single functional module propagates through interconnected mitochondrial networks. Consequently, metabolic inflexibility emerges as a systems-level manifestation of coordinated mitochondrial remodeling rather than the isolated consequence of defective lipid metabolism [65].

Experimental studies consistently demonstrate that interventions preserving mitochondrial lipid metabolism are associated with improved metabolic flexibility and enhanced mitochondrial performance. Among these, curcumin represents a well-characterized experimental example that maintains the expression of proteins involved in mitochondrial fatty acid transport and β-oxidation while improving metabolic adaptation under chronic metabolic stress [33]. These observations support the concept that restoration of metabolic flexibility is more closely associated with stabilization of coordinated metabolic networks than with regulation of individual enzymes participating in FAO.

Within the MCBN framework, preservation of FAO contributes not only to efficient lipid utilization but also to maintenance of metabolic flexibility, mitochondrial adaptability, and coordinated communication among bioenergetic, redox, and adaptive signaling networks [47]. Accordingly, restoration of metabolic flexibility should be interpreted as a systems-level indicator of improved mitochondrial homeostasis rather than simply enhanced fatty acid catabolism.

### 5.4. Redox Consequences: Oxidative Stress as a Network-Level Phenomenon

Disruption of redox homeostasis represents one of the earliest and most pervasive functional consequences of mitochondrial network remodeling during metabolic disease. Because mitochondrial ROS participate in both oxidative metabolism and intracellular signaling, maintenance of redox balance is essential for preserving mitochondrial adaptability under conditions of chronic metabolic stress. Comparative proteomic and functional studies consistently demonstrate that coordinated remodeling of mitochondrial redox pathways is associated with impaired antioxidant capacity, altered redox signaling, and progressive loss of cellular resilience [38,46,66].

Importantly, oxidative stress should not be interpreted solely as excessive ROS accumulation. Contemporary redox biology recognizes that physiological ROS function as essential signaling mediators regulating mitochondrial biogenesis, metabolic adaptation, inflammatory responses, and cell survival. Consequently, disruption of redox homeostasis simultaneously compromises antioxidant defense mechanisms and the signaling processes required for mitochondrial adaptation. Emerging evidence from redox proteomics further indicates that metabolic stress remodels redox-sensitive PTMs, providing an additional regulatory layer through which mitochondrial functional networks are dynamically reorganized [67,68].

Experimental studies consistently demonstrate that interventions capable of improving mitochondrial redox balance are also associated with preservation of mitochondrial function and enhanced adaptive capacity. Curcumin attenuates oxidative stress while maintaining antioxidant defense systems and improving mitochondrial redox homeostasis. These findings support the interpretation that restoration of redox resilience reflects stabilization of coordinated mitochondrial signaling networks rather than simple scavenging of ROS.

Within the proposed MCBN framework, redox homeostasis functions as an organizational interface linking mitochondrial bioenergetics, metabolic regulation, proteostasis, and inflammatory signaling. Preservation of redox resilience therefore supports not only protection against oxidative damage but also maintenance of the coordinated communication required for adaptive mitochondrial responses.

### 5.5. Mitochondrial Proteostasis and Adaptive Capacity

Maintenance of mitochondrial proteostasis is essential for preserving the adaptive capacity of MCBNs during chronic metabolic stress. Rather than functioning solely as a quality-control system, mitochondrial proteostasis maintains the structural and functional integrity of the mitochondrial proteome through coordinated regulation of protein folding, import, proteolytic turnover, the UPR^mt^, mitophagy, and other quality-control mechanisms [24]. Comparative proteomic studies consistently indicate that disruption of these interconnected processes accompanies obesity, T2D, and chronic nutrient excess, suggesting that impaired proteostasis contributes directly to progressive mitochondrial dysfunction.

Progressive loss of proteostatic capacity has functional consequences that extend far beyond the accumulation of damaged proteins. Failure to maintain protein integrity compromises OXPHOS, metabolic flexibility, redox homeostasis, and mitochondrial signaling, thereby weakening communication among interconnected functional modules. Consequently, proteostatic dysfunction reduces the ability of mitochondria to respond to persistent metabolic stress, accelerating loss of cellular resilience and promoting progressive network destabilization [40].

Experimental studies consistently demonstrate that interventions preserving mitochondrial protein quality-control mechanisms are associated with improved mitochondrial function and enhanced adaptive capacity. These observations support the interpretation that functional recovery depends on stabilization of the proteostasis module as an integrated component of mitochondrial network organization rather than selective regulation of individual chaperones or proteases.

Within the MCBN framework, mitochondrial proteostasis represents the structural maintenance system that preserves the integrity, adaptability, and coordinated function of interconnected mitochondrial biological networks. Accordingly, preservation of proteostatic capacity should be interpreted as a systems-level indicator of mitochondrial resilience and an essential component of mitochondrial homeostasis during chronic metabolic disease.

### 5.6. Inflammation and the Propagation of Network Dysfunction

Activation of inflammatory signaling represents one of the principal mechanisms through which mitochondrial network dysfunction extends beyond the organelle to influence cellular and tissue physiology. Increasing evidence indicates that mitochondrial dysfunction and inflammation should not be interpreted as independent pathological processes but as interconnected components of a self-amplifying adaptive response to chronic metabolic stress [69,70]. Comparative proteomic and functional studies consistently demonstrate that progressive disruption of mitochondrial bioenergetics, metabolic regulation, redox homeostasis, and proteostasis is accompanied by activation of inflammatory pathways that further compromise mitochondrial function.

Among the signaling pathways most consistently associated with metabolic disease progression are NF-κB, JNK, p38 mitogen-activated protein kinase (p38 MAPK) and the NLRP3 inflammasome [4,71,72]. Coordinated activation of these signaling networks promotes production of pro-inflammatory mediators, including tumor necrosis factor-alpha (TNF-α), interleukin-1 beta (IL-1β), interleukin-6 (IL-6) and monocyte chemoattractant protein-1 (MCP-1), thereby reinforcing oxidative stress, impairing insulin signaling, and accelerating tissue remodeling. Rather than functioning independently, these inflammatory pathways participate in reciprocal interactions with mitochondrial functional modules, creating positive feedback loops that progressively reduce mitochondrial adaptability.

Experimental studies consistently demonstrate that interventions capable of improving mitochondrial function are also associated with attenuation of inflammatory activation and preservation of tissue physiology. Among these, curcumin represents a well-characterized experimental example that reduces activation of inflammatory signaling while simultaneously improving mitochondrial bioenergetics, redox balance, and metabolic adaptation. These observations support the concept that modulation of inflammatory responses reflects stabilization of interconnected mitochondrial biological networks rather than selective inhibition of individual inflammatory mediators [73].

Within the proposed MCBN framework, inflammation is best interpreted as a network-level consequence of progressive mitochondrial instability rather than as an isolated pathological pathway. Once established, inflammatory signaling further destabilizes mitochondrial bioenergetic, metabolic, redox, and proteostatic networks, generating a self-reinforcing cycle that progressively diminishes tissue resilience. Accordingly, limitation of inflammatory amplification represents a systems-level indicator of restored mitochondrial homeostasis rather than merely suppression of inflammatory signaling.

### 5.7. Fibrotic Remodeling as a Terminal Manifestation of Network Failure

Fibrotic remodeling represents one of the most advanced functional consequences of persistent mitochondrial network destabilization during chronic metabolic disease. Although fibrosis is often regarded as a tissue-specific pathological process, increasing evidence indicates that common profibrotic mechanisms operate across metabolically active organs, including the kidney, liver, and heart [74]. Comparative experimental studies consistently associate prolonged mitochondrial dysfunction, oxidative stress, and chronic inflammatory activation with progressive extracellular matrix remodeling and irreversible deterioration of tissue architecture.

From the perspective of the MCBN framework, fibrotic remodeling should be interpreted as the cumulative consequence of sustained failure of mitochondrial adaptation rather than activation of an isolated profibrotic pathway. Progressive impairment of bioenergetic, metabolic, redox, proteostatic, and inflammatory modules gradually reduces tissue resilience, creating a biological environment that favors activation of TGF-β, Smad-dependent signaling, and extracellular matrix deposition [75,76,77,78,79]. Consequently, fibrosis emerges as the structural manifestation of prolonged mitochondrial network instability.

Experimental studies consistently demonstrate that interventions preserving mitochondrial function are associated with attenuation of fibrotic remodeling and improved tissue integrity. Among these, curcumin represents an experimental example that simultaneously reduces oxidative stress, inflammatory activation, and extracellular matrix accumulation while preserving multiple mitochondrial functional modules. These findings support the interpretation that limitation of fibrosis is more closely associated with stabilization of upstream mitochondrial biological networks than with selective inhibition of individual profibrotic mediators.

Within the MCBN framework, fibrotic remodeling represents the terminal structural phenotype resulting from persistent loss of mitochondrial homeostasis. Accordingly, preservation of mitochondrial network stability may delay or limit the transition from reversible functional adaptation to irreversible tissue remodeling. This interpretation places fibrosis within a continuum of progressive mitochondrial dysfunction rather than as an independent endpoint of metabolic disease.

### 5.8. Curcumin as a Representative Systems-Level Modulator of Mitochondrial Homeostasis

From the perspective of the MCBN framework, effective interventions are expected to preserve mitochondrial homeostasis through coordinated modulation of multiple interconnected functional modules rather than selective regulation of individual molecular targets. This systems-level concept is consistent with the growing recognition that complex metabolic diseases arise from network dysfunction and therefore require interventions capable of restoring integrated mitochondrial function rather than correcting isolated biochemical abnormalities [80].

Among the experimental interventions reviewed herein, curcumin represents one of the best-characterized examples of this systems-level behavior. Comparative studies consistently demonstrate coordinated preservation of mitochondrial bioenergetics, FAO, redox homeostasis, proteostasis, inflammatory regulation, and limitation of fibrotic remodeling across multiple experimental models [33,80,81]. Collectively, these observations suggest that the biological effects of curcumin are better explained by modulation of interconnected mitochondrial functional modules than by selective regulation of any single signaling pathway.

The capacity to influence multiple mitochondrial functional modules simultaneously may help explain why curcumin consistently demonstrates protective effects across diverse organs and experimental models despite substantial biological heterogeneity. Rather than functioning as a pathway-specific intervention, curcumin illustrates how preservation of mitochondrial network organization can generate coordinated improvements in bioenergetic performance, metabolic flexibility, redox resilience, proteostatic capacity, inflammatory regulation, and tissue integrity [12].

Accordingly, the biological actions of curcumin should be interpreted as representative evidence supporting the broader concept that preservation of mitochondrial homeostasis emerges from coordinated stabilization of interconnected biological networks. Although additional pharmacological and non-pharmacological interventions remain to be investigated within this framework, the available evidence illustrates how network-oriented modulation may provide a more comprehensive strategy for improving mitochondrial adaptation during obesity, T2D, and chronic nutrient excess [82].

### 5.9. Functional Integration Supports the Mitochondrial Homeostasis Hypothesis

Viewed collectively, the proteomic, bioenergetic, biochemical, and physiological evidence reviewed throughout this article reveals a remarkably consistent pattern of mitochondrial adaptation during metabolic disease. Across independent experimental models, coordinated remodeling of conserved mitochondrial functional modules is reproducibly associated with impaired bioenergetic resilience, loss of metabolic flexibility, disruption of redox homeostasis, reduced proteostatic capacity, inflammatory amplification, and progressive tissue remodeling. Rather than representing independent pathological events, these functional alterations emerge as interconnected manifestations of mitochondrial network destabilization. This functional convergence provides a compelling biological rationale for interpreting mitochondrial homeostasis as an emergent systems-level property arising from coordinated regulation of interconnected mitochondrial functional networks. Individual proteins and signaling pathways undoubtedly contribute to this process; however, the reproducible preservation or disruption of integrated functional modules across diverse tissues and experimental conditions offers a more comprehensive explanation for mitochondrial adaptation than isolated molecular mechanisms [32]. The MCBN framework therefore provides a conceptual bridge linking proteomic remodeling with functional outcomes observed during metabolic disease progression.

Accordingly, the Mitochondrial Homeostasis Hypothesis should be viewed as a testable conceptual model derived from the convergence of comparative proteomic and functional evidence rather than as a definitive mechanistic explanation [33]. This framework proposes that mitochondrial homeostasis emerges from the coordinated behavior of interconnected biological networks whose preservation determines cellular resilience under chronic metabolic stress.

## 6. Regulatory Architecture of Mitochondrial-Centered Biological Networks

### 6.1. Molecular Regulation of Mitochondrial Homeostasis

Sustained therapeutic benefit is more likely to arise from coordinated stabilization of mitochondrial functional networks than from selective modulation of individual molecular targets. This emerging concept provides the biological rationale for precision mitochondrial medicine and establishes the conceptual foundation for interpreting mitochondrial signaling pathways as components of an integrated regulatory architecture rather than isolated molecular cascades.

Maintenance of mitochondrial homeostasis requires continuous integration of information regarding nutrient availability, energetic status, oxidative stress, inflammatory stimuli, and environmental challenges. Rather than being governed by isolated signaling pathways, this adaptive process depends on a highly interconnected regulatory architecture that coordinates mitochondrial metabolism, biogenesis, proteostasis, redox homeostasis, and cellular resilience [33,43,44]. Within the MCBN framework, signaling pathways should therefore be interpreted as dynamic regulatory modules whose coordinated interactions determine the adaptive behavior of MCBNs [12].

The proteomic and functional evidence reviewed in previous sections indicates that preservation of mitochondrial homeostasis depends on the dynamic balance between adaptive and maladaptive regulatory modules. Adaptive pathways-including AMPK, SIRT1, PGC-1α, PPARα and PPARγ, and Nrf2-promote mitochondrial metabolism, metabolic flexibility, redox resilience, and cellular adaptation, whereas persistent activation of NF-κB, JNK, and TGF-β progressively destabilizes these functions during chronic metabolic stress. Collectively, these signaling modules constitute an integrated regulatory network that governs mitochondrial resilience and disease progression.

Representative experimental interventions, including curcumin, illustrate how simultaneous modulation of multiple regulatory modules is associated with preservation of mitochondrial homeostasis [47]. These observations support the interpretation that mitochondrial adaptation is more effectively achieved through coordinated regulation of interconnected signaling networks than through selective manipulation of individual molecular pathways.

### 6.2. The AMPK–SIRT1–PGC-1α Axis: Master Adaptive Regulatory Module of Mitochondrial Homeostasis

Among the regulatory modules governing mitochondrial adaptation, the AMPK–SIRT1–PGC-1α axis represents the principal integrative network linking cellular energetic status with long-term mitochondrial remodeling. Rather than functioning as a linear signaling cascade, this interconnected regulatory module coordinates transcriptional and metabolic responses that preserve mitochondrial adaptability during energetic and metabolic stress [83,84]. Within the proposed MCBN framework, this axis functions as the master adaptive regulatory module responsible for synchronizing multiple mitochondrial functional networks [85].

AMPK senses reductions in cellular energy availability and initiates adaptive responses that restore energetic balance. SIRT1 complements this process by coupling mitochondrial metabolic activity to NAD^+^-dependent transcriptional regulation, whereas PGC-1α integrates these upstream signals into coordinated transcriptional programs governing mitochondrial biogenesis, oxidative metabolism, fatty acid utilization, antioxidant defenses, and metabolic adaptation [43]. Rather than acting independently, these regulators function as an integrated adaptive module whose coordinated activity preserves mitochondrial resilience.

From a systems perspective, activation of the AMPK-SIRT1-PGC-1α axis simultaneously reinforces multiple mitochondrial functional modules previously discussed in this review, including bioenergetic resilience, metabolic flexibility, redox homeostasis, proteostatic capacity, and adaptive stress responses. Conversely, progressive impairment of this regulatory module compromises coordinated network behavior, contributing to the functional deterioration observed during obesity, T2D, and chronic nutrient excess [3,64]. In this context, regulatory modules govern the dynamic behavior of the mitochondrial functional modules revealed by comparative proteomic analyses, thereby providing the mechanistic framework through which proteomic remodeling is translated into coordinated functional adaptation.

Representative experimental evidence indicates that curcumin enhances several components of this adaptive regulatory module, including activation of AMPK, increased SIRT1 activity, and enhanced PGC-1α signaling [86,87]. These coordinated effects are consistently associated with improvements in mitochondrial respiration, metabolic adaptation, and tissue resilience. Accordingly, curcumin provides representative evidence that preservation of the AMPK–SIRT1–PGC-1α regulatory network supports maintenance of mitochondrial homeostasis through integrated regulation of interconnected mitochondrial functional modules [81].

### 6.3. PPAR Signaling and the Maintenance of Metabolic Flexibility

Maintenance of metabolic flexibility requires continuous coordination between substrate availability, mitochondrial oxidative capacity, and systemic energy demands. Within the proposed MCBN framework, PPAR-dependent signaling constitutes the principal regulatory module governing this adaptive process by integrating mitochondrial lipid metabolism with whole-body metabolic homeostasis. Rather than functioning as independent nuclear receptors, PPARα and PPARγ orchestrate complementary transcriptional programs that preserve metabolic adaptability during chronic energetic stress [88,89,90].

PPARα predominantly regulates mitochondrial FAO and oxidative substrate utilization, whereas PPARγ coordinates insulin sensitivity, lipid partitioning, adipose tissue function, and inflammatory balance. Together, these complementary activities synchronize cellular substrate utilization with tissue metabolic demands, thereby preserving mitochondrial metabolic efficiency and systemic metabolic flexibility.

From a systems perspective, PPAR-dependent signaling regulates one of the central functional modules identified throughout this review: metabolic flexibility [90,91]. Progressive impairment of this regulatory network reduces the capacity to switch efficiently between metabolic substrates, thereby promoting lipid accumulation, mitochondrial dysfunction, insulin resistance, and progressive destabilization of interconnected mitochondrial functional networks [92]. Accordingly, PPAR signaling provides the regulatory control through which proteomic remodeling of lipid metabolic pathways is translated into coordinated metabolic adaptation.

### 6.4. Nrf2 and the Preservation of Redox Network Stability

Preservation of redox resilience is essential for maintaining mitochondrial adaptability during chronic metabolic stress. Rather than simply limiting oxidative damage, redox regulation coordinates physiological signaling, metabolic adaptation, proteostasis, and bioenergetic performance [93,94]. Within the proposed MCBN framework, Nrf2 functions as the principal adaptive regulatory module governing this integrated redox network.

Under physiological conditions, Nrf2 remains associated with Keap1 in the cytoplasm. Oxidative or electrophilic stimuli promote dissociation of this complex, allowing Nrf2 to activate transcriptional programs that enhance antioxidant defenses, glutathione metabolism, thioredoxin systems, peroxiredoxins, and other cytoprotective mechanisms [95,96]. Collectively, these responses preserve physiological redox signaling while preventing the accumulation of irreversible oxidative damage.

From a systems perspective, Nrf2 regulates the functional redox module identified throughout this review by coordinating antioxidant defenses with mitochondrial bioenergetics, metabolic adaptation, proteostatic capacity, and redox-sensitive signaling pathways. Emerging evidence from comparative proteomics and redox proteomics further indicates that this regulatory module influences reversible PTMs that contribute to coordinated mitochondrial adaptation during metabolic stress. Accordingly, Nrf2 provides the regulatory framework through which redox-sensitive proteomic remodeling is translated into preservation of mitochondrial redox resilience, thereby linking molecular signaling with systems-level mitochondrial adaptation.

Collectively, these observations support the concept that Nrf2 functions as a conserved regulatory module that preserves mitochondrial redox homeostasis and contributes to adaptive network stability across metabolically active tissues.

### 6.5. NF-κB and Maladaptive Network Signaling

Progressive destabilization of MCBNs requires amplification mechanisms capable of propagating local mitochondrial dysfunction into widespread cellular and tissue responses. Within the MCBN framework, NF-κB represents the principal maladaptive regulatory module responsible for this transition by integrating inflammatory signaling with metabolic dysfunction, oxidative stress, and impaired mitochondrial adaptation.

Persistent activation of NF-κB promotes sustained production of pro-inflammatory cytokines, including TNF-α, IL-1β, IL-6, and MCP-1, establishing self-reinforcing signaling circuits that impair mitochondrial metabolism, enhance oxidative stress, and progressively reduce cellular adaptive capacity [4]. Increasing evidence indicates that Nrf2 and NF-κB do not operate as independent signaling pathways but rather constitute functionally interconnected regulatory modules whose reciprocal interactions determine the balance between adaptive and maladaptive cellular responses [97]. Rather than functioning as an isolated inflammatory pathway, NF-κB amplifies reciprocal interactions among bioenergetic, metabolic, redox, and proteostatic dysfunction, thereby accelerating network destabilization during obesity, T2D, and chronic nutrient excess.

From a systems perspective, the reciprocal balance between adaptive regulators such as Nrf2 and maladaptive modules such as NF-κB determines whether mitochondrial networks maintain resilience or progress toward chronic dysfunction. Loss of adaptive signaling permits inflammatory amplification, whereas restoration of regulatory balance limits propagation of network instability and favors recovery of coordinated mitochondrial function. Accordingly, NF-κB provides the principal regulatory mechanism through which inflammatory amplification is translated into progressive mitochondrial network destabilization.

Representative experimental studies consistently demonstrate attenuation of NF-κB activation following curcumin treatment across diverse models of metabolic disease [14,17]. Together with the simultaneous activation of adaptive regulatory pathways, these observations suggest that curcumin contributes to restoration of regulatory network balance rather than selective inhibition of inflammatory signaling [81]. Accordingly, curcumin provides representative evidence that limiting maladaptive signaling supports preservation of coordinated mitochondrial adaptation within the proposed MCBN framework.

### 6.6. Crosstalk Between Adaptive and Maladaptive Signaling Networks

One of the central concepts emerging from the proposed MCBN framework is that mitochondrial adaptation depends on the dynamic balance established among interconnected regulatory modules rather than on the activity of individual signaling pathways [32]. Adaptive responses arise from coordinated interactions among multiple signaling networks whose collective behavior determines mitochondrial resilience under changing metabolic conditions.

Adaptive regulatory modules-including AMPK, SIRT1, PGC-1α, PPARs, and Nrf2-collectively reinforce mitochondrial bioenergetics, metabolic flexibility, proteostatic capacity, and redox resilience. Conversely, persistent activation of maladaptive modules such as NF-κB, JNK, and TGF-β progressively amplifies inflammatory signaling, oxidative stress, metabolic dysfunction, and tissue remodeling. The dynamic balance established between these opposing regulatory influences ultimately determines the adaptive state of MCBNs. Progressive disruption of this balance shifts mitochondrial regulation from adaptive toward maladaptive network states, thereby driving the functional deterioration associated with chronic metabolic disease.

From a systems perspective, progression from metabolic health to obesity and T2D can therefore be interpreted as a gradual transition from adaptive toward maladaptive regulatory states rather than abrupt activation of isolated pathological pathways. As adaptive regulatory capacity declines, reciprocal reinforcement among inflammatory, metabolic, and redox disturbances promotes self-sustaining network destabilization, progressively reducing mitochondrial resilience and tissue adaptability. Within this framework, mitochondrial homeostasis is not a static condition but an emergent dynamic regulatory state resulting from continuous coordination among adaptive and maladaptive MCBNs. Accordingly, mitochondrial homeostasis should be interpreted as a dynamic systems-level property maintained through continuous rebalancing of interconnected regulatory networks.

Representative experimental evidence indicates that curcumin simultaneously enhances adaptive regulatory modules while attenuating maladaptive signaling pathways across diverse models of metabolic disease [81]. Rather than acting through a single dominant molecular target, these coordinated effects illustrate how restoration of regulatory network balance can promote recovery of mitochondrial adaptation. Collectively, these observations support the concept that preservation of mitochondrial homeostasis depends on coordinated regulation of interconnected signaling networks rather than selective modulation of individual pathways [81].

### 6.7. Signaling Integration Supports the Mitochondrial Homeostasis Hypothesis

Collectively, the regulatory modules discussed throughout this section define an integrated signaling architecture that governs mitochondrial adaptation under physiological and pathological conditions [33]. Rather than functioning as independent molecular pathways, adaptive and maladaptive signaling modules interact dynamically to coordinate mitochondrial bioenergetics, metabolic flexibility, redox resilience, proteostatic capacity, inflammatory regulation, and tissue remodeling. This integrated regulatory organization provides the mechanistic basis for interpreting mitochondrial function as an emergent systems-level property rather than the product of isolated molecular events.

The convergence of comparative proteomic, functional, and regulatory evidence reviewed throughout this article consistently supports the interpretation that mitochondrial homeostasis emerges from coordinated interactions among interconnected MCBNs [12]. Within this framework, signaling pathways do not operate as independent determinants of cellular adaptation but as regulatory modules that govern the dynamic behavior of the conserved functional modules revealed through comparative proteomic analyses. Accordingly, mitochondrial homeostasis should be interpreted as the emergent consequence of regulatory coordination rather than the isolated activity of individual signaling pathways.

Taken together, the regulatory architecture described in this section provides the mechanistic foundation for the Mitochondrial Homeostasis Hypothesis developed in the following section. This hypothesis proposes that mitochondrial homeostasis represents an emergent systems-level property arising from coordinated interactions among bioenergetic, metabolic, redox, proteostatic, inflammatory, and regulatory mitochondrial networks. Consequently, preservation of mitochondrial adaptation, and ultimately the development of precision mitochondrial medicine, should be viewed as the result of maintaining coordinated network organization rather than selective regulation of individual molecular pathways.

## 7. Redox Regulation Within Mitochondrial-Centered Biological Networks

### 7.1. Redox Homeostasis as a Systems-Level Property

Although this definition remains useful, contemporary redox biology demonstrates that cellular redox regulation is considerably more dynamic and complex than originally appreciated [38,98]. Rather than acting solely as damaging by-products of metabolism, ROS function as essential signaling molecules that regulate gene expression, metabolic adaptation, mitochondrial communication, stress responses, and cell fate decisions [39,99]. Moreover, recent advances in redox proteomics have revealed that many of these regulatory effects are mediated through reversible oxidative PTMs that dynamically modulate protein function rather than simply reflecting irreversible oxidative damage [68].

From a systems biology perspective, redox homeostasis emerges as an integrated systems-level property resulting from the coordinated interactions among mitochondrial metabolism, antioxidant defense systems, adaptive signaling pathways, and protein quality-control mechanisms. Within this framework, preservation of redox resilience depends not only on limiting excessive ROS accumulation but also on maintaining physiological redox signaling and the capacity of cells to dynamically adapt to metabolic and environmental stress [46]. Consequently, oxidative stress is better understood as a manifestation of progressive network instability that develops when the adaptive regulatory capacity of MCBNs is exceeded [94].

This systems-level interpretation is particularly relevant in obesity, T2D, and hypercaloric diet-induced metabolic dysfunction, where multiple mitochondrial functional modules become simultaneously perturbed. As discussed in previous sections, alterations affecting OXPHOS, FAO, mitochondrial proteostasis, and adaptive signaling directly influence ROS generation, redox-sensitive PTMs, and mitochondrial communication, thereby promoting progressive destabilization of MCBNs. Accordingly, preservation of redox homeostasis represents both a determinant and a consequence of mitochondrial network stability.

### 7.2. Mitochondrial ROS Production During Metabolic Stress

Mitochondria constitute the principal intracellular source of ROS under both physiological and pathological conditions [39]. During OXPHOS, a small proportion of electrons escaping from the respiratory chain react with molecular oxygen to generate superoxide. Under physiological conditions, these ROS act as signaling mediators that contribute to metabolic adaptation, mitochondrial communication, and cellular stress responses without causing significant cellular injury [35,100].

Metabolic stress profoundly alters this physiological balance. Obesity, T2D, and chronic exposure to hypercaloric diets increase substrate availability and the generation of reducing equivalents, thereby enhancing electron transport activity and increasing the probability of electron leakage [3,10]. As ROS production progressively exceeds adaptive buffering capacity, physiological redox signaling shifts toward persistent oxidative stress, leading to widespread oxidative PTMs that alter enzyme activity, signaling pathways, and mitochondrial function [55].

Rather than representing isolated biochemical events, excessive ROS production initiates coordinated remodeling of multiple mitochondrial functional modules. Oxidative modifications impair OXPHOS, disrupt proteostasis, alter metabolic signaling, and progressively reduce bioenergetic efficiency while simultaneously amplifying inflammatory and redox-sensitive signaling pathways [39,99]. These reciprocal interactions establish self-reinforcing feedback loops that promote progressive destabilization of MCBNs.

Within the proposed MCBN framework, chronic metabolic disease may therefore be interpreted as a progressive transition from adaptive redox signaling toward maladaptive oxidative network states. Accordingly, excessive mitochondrial ROS production represents not merely a biochemical consequence of metabolic overload but a systems-level mechanism driving the loss of mitochondrial homeostasis and tissue resilience during chronic metabolic disease.

### 7.3. Proteomic Evidence of Redox Network Remodeling

One of the most reproducible observations emerging from comparative proteomic studies is the recurrent identification of proteins involved in redox regulation and antioxidant defense across multiple tissues and experimental models of metabolic disease. Rather than representing isolated molecular alterations, these findings consistently indicate coordinated remodeling of a conserved redox functional module within MCBNs. Proteins belonging to the peroxiredoxin, thioredoxin, glutathione, and superoxide dismutase systems are among the most frequently identified components of this adaptive network [38,66].

Importantly, recent advances in redox proteomics indicate that remodeling of these networks extends beyond changes in protein abundance. Recent advances in redox proteomics demonstrate that reversible oxidative PTMs, including thiol oxidation, S-glutathionylation, S-nitrosylation, and sulfenylation, dynamically regulate the activity, localization, and molecular interactions of numerous mitochondrial proteins [68,101,102]. Consequently, remodeling of MCBNs occurs through coordinated quantitative and qualitative changes in proteome organization, providing an additional regulatory layer that links protein abundance with protein function.

Consistent with these observations, pathway enrichment analyses repeatedly identify biological processes related to oxidative stress responses, ROS metabolism, cellular detoxification, and antioxidant defense among the most significantly altered functional categories in obesity and T2D models. The remarkable reproducibility of these findings across independent datasets provides convergent evidence that redox homeostasis represents a conserved functional module of MCBNs rather than an isolated biochemical process.

From a systems biology perspective, the recurrent identification of redox-associated proteins and redox-sensitive PTMs indicates that oxidative remodeling should not be interpreted merely as a downstream consequence of metabolic dysfunction. Instead, coordinated remodeling of redox regulatory networks contributes directly to mitochondrial communication, adaptive signaling, and network behavior. Accordingly, preservation of redox network integrity emerges as a critical determinant of mitochondrial homeostasis and tissue resilience during chronic metabolic stress.

### 7.4. Carbonyl Stress and Glycation of Mitochondrial Proteins

In addition to classical oxidative stress, metabolic diseases promote carbonyl stress, characterized by accumulation of reactive carbonyl species and the non-enzymatic modification of proteins [101]. This phenomenon is particularly relevant under conditions of high fructose consumption because fructose exhibits substantially greater glycation potential than glucose [103,104]. Carbonyl stress contributes to the formation of advanced glycation end products, leading to structural and functional alterations that affect mitochondrial metabolism, inflammatory signaling, and cellular adaptation.

Studies performed by our research group demonstrated that fructose-induced glycation preferentially targets proteins involved in OXPHOS, cellular respiration and TCA cycle activity, providing direct evidence that key components of mitochondrial metabolism are highly susceptible to carbonyl-mediated remodeling during metabolic disease progression [54]. Unlike reversible oxidative PTMs, glycation generally represents a more persistent modification capable of progressively impairing enzyme activity, protein–protein interactions, and metabolic communication.

Representative experimental studies demonstrate that curcumin attenuates protein glycation and carbonyl stress across multiple models of metabolic dysfunction [85]. Rather than supporting a compound-specific mechanism, these observations illustrate how preservation of mitochondrial protein integrity contributes to stabilization of interconnected mitochondrial networks, providing representative evidence for the broader principles underlying the Mitochondrial Homeostasis Hypothesis.

### 7.5. Redox Proteomics: Expanding the Understanding of MCBNs

Although conventional proteomics provides comprehensive information regarding changes in protein abundance, it offers only limited insight into the oxidative PTMs that directly regulate protein function. Redox proteomics extends this capability by enabling the identification and quantification of reversible and irreversible oxidative modifications affecting specific proteins, thereby providing a second layer of functional information about the mitochondrial proteome [68,98,101,105]. Rather than simply cataloging protein expression, redox proteomics characterizes how oxidative PTMs dynamically influence enzyme activity, protein stability, molecular interactions, and signaling capacity.

Among the most biologically relevant oxidative PTMs are protein carbonylation, S-nitrosylation, S-glutathionylation, and cysteine sulfenylation [102]. These modifications regulate numerous mitochondrial proteins involved in bioenergetics, metabolism, antioxidant defense, and proteostasis. Because many mitochondrial proteins are near ROS-generating sites within the respiratory chain, they are particularly susceptible to redox-dependent functional remodeling. Consequently, oxidative PTMs constitute a dynamic regulatory layer that complements quantitative proteomic remodeling and determines the functional state of conserved mitochondrial modules.

Within the MCBN framework, redox proteomics provides the mechanistic link between quantitative proteomic remodeling and systems-level mitochondrial adaptation. Comparative proteomics identifies the conserved functional modules undergoing remodeling, whereas redox proteomics reveals how reversible oxidative PTMs dynamically regulate the activity of those modules in response to metabolic stress. Together, these complementary approaches offer a more comprehensive understanding of how MCBNs adapt, or progressively lose adaptive capacity, during obesity and T2D.

Future application of integrated quantitative and redox proteomics to experimental models of obesity, diabetes, and therapeutic intervention is expected to provide critical mechanistic insight into the dynamic regulation of MCBNs [46]. Beyond identifying oxidative damage, these approaches may distinguish adaptive redox signaling from persistent oxidative injury and contribute to experimental validation of the Mitochondrial Homeostasis Hypothesis by revealing how coordinated changes in protein abundance and oxidative PTMs collectively determine mitochondrial network stability [46,68,101].

### 7.6. Nrf2 as the Principal Regulator of Redox Adaptation

Among the signaling pathways governing cellular antioxidant responses, Nrf2 functions as the principal adaptive regulator of redox resilience [46,93]. Activation of Nrf2 induces expression of genes involved in glutathione synthesis, thioredoxin metabolism, peroxiredoxin activity, heme oxygenase-1 expression, and NAD(P)H quinone oxidoreductase activity, thereby strengthening multiple interconnected antioxidant defense systems rather than isolated enzymatic pathways [94].

From a systems biology perspective, Nrf2 should be viewed not simply as an antioxidant pathway but as the central regulatory module coordinating the redox component of MCBNs. Through integration of mitochondrial bioenergetics, metabolic adaptation, proteostasis, and stress-responsive signaling, Nrf2 preserves physiological redox signaling while enhancing cellular resilience during chronic metabolic stress. Consequently, maintenance of Nrf2 activity contributes to preservation of adaptive network behavior rather than antioxidant defense alone.

Representative experimental studies consistently demonstrate activation of Nrf2 signaling together with enhanced expression of antioxidant defense proteins following curcumin treatment [95,96]. These coordinated molecular responses are associated with preservation of mitochondrial respiration, maintenance of physiological redox signaling, and improved tissue adaptation. Accordingly, curcumin provides representative evidence that stabilization of the Nrf2 regulatory module contributes to preservation of mitochondrial redox resilience, illustrating how coordinated regulation of adaptive signaling networks supports systems-level mitochondrial adaptation [81].

### 7.7. Crosstalk Between Redox and Inflammatory Networks

One of the most important concepts emerging from contemporary redox biology is that oxidative stress and inflammation constitute highly interconnected regulatory networks that collectively govern cellular adaptation [46]. Rather than representing independent pathological processes, redox and inflammatory signaling communicate through multiple reciprocal feedback mechanisms that dynamically influence mitochondrial function, tissue homeostasis, and disease progression [99,106]. Within this regulatory architecture, mitochondria function not only as mediators of oxidative damage but also as signaling molecules that coordinate communication among interconnected biological networks [100].

Among the most important regulatory interactions is the reciprocal crosstalk between Nrf2 and NF-κB signaling [97]. Under physiological conditions, Nrf2 promotes preservation of adaptive redox signaling and mitochondrial resilience, whereas persistent NF-κB activation amplifies inflammatory responses and progressively suppresses mitochondrial adaptation. Disruption of this regulatory balance establishes self-reinforcing feedback loops linking oxidative stress, inflammation, and mitochondrial dysfunction, thereby driving progressive destabilization of MCBNs.

From a systems perspective, chronic metabolic disease can therefore be interpreted as a progressive failure to preserve coordinated communication between adaptive redox and inflammatory regulatory modules. Accordingly, the interaction between Nrf2 and NF-κB represents a regulatory interface through which local mitochondrial dysfunction is amplified into widespread metabolic and inflammatory disturbances, promoting loss of tissue resilience and progressive network instability.

Representative experimental studies illustrate this systems-level interaction. Hypercaloric diet exposure was associated with simultaneous increases in oxidative stress, inflammatory activation, and tissue injury, supporting coordinated remodeling of interconnected redox and inflammatory networks. Curcumin attenuated these responses concurrently, providing representative evidence that restoration of regulatory network balance contributes to stabilization of MCBNs rather than selective modulation of individual signaling pathways [81].

### 7.8. Fibrosis as a Downstream Consequence of Redox Network Failure

Persistent disruption of redox homeostasis contributes not only to oxidative damage but also to progressive extracellular matrix remodeling and fibrosis through activation of multiple redox-sensitive signaling pathways [74]. Within the MCBN framework, fibrosis should therefore be interpreted as a downstream systems-level consequence of prolonged mitochondrial network instability rather than an isolated pathological process. Oxidative stress regulates fibroblast activation, extracellular matrix deposition, and tissue remodeling through multiple interconnected signaling mechanisms that include TGF-β, Smad proteins, MAPKs, and other redox-sensitive profibrotic pathways [77,78,107].

From a systems biology perspective, fibrotic remodeling emerges from the cumulative effects of sustained disturbances affecting bioenergetic, metabolic, redox, inflammatory, and proteostatic networks. As MCBNs progressively lose adaptive capacity, profibrotic signaling becomes increasingly self-sustaining, ultimately promoting irreversible structural remodeling and reduced tissue resilience. Accordingly, fibrosis represents the terminal structural phenotype of persistent mitochondrial network destabilization rather than the consequence of dysregulation within a single signaling pathway.

Representative experimental evidence obtained in our renal studies illustrates this systems-level progression. Hypercaloric diet-induced oxidative stress and inflammatory activation occurred in parallel with increased fibrotic remodeling, supporting coordinated deterioration of interconnected biological networks. Curcumin simultaneously attenuated oxidative stress, inflammation, and fibrosis, suggesting restoration of upstream mitochondrial network stability rather than direct inhibition of profibrotic pathways alone [81]. Similar relationships have been reported in liver and cardiac models, where improvements in mitochondrial function are consistently associated with attenuation of tissue fibrosis and preservation of organ architecture [77,78].

Collectively, these observations support the interpretation that preservation of mitochondrial network stability may delay or prevent the transition from reversible adaptive remodeling to irreversible fibrotic remodeling. Within the proposed MCBN framework, fibrosis therefore represents the structural endpoint of progressive network failure, reinforcing the concept that maintenance of mitochondrial homeostasis is fundamentally linked to preservation of coordinated biological network integrity.

### 7.9. Curcumin Preserves Redox Network Integrity and Prevents MCBNs Collapse

A systems-level perspective reveals that redox regulation extends far beyond the control of ROS. Within the MCBNs, redox processes function as integrative mechanisms that coordinate mitochondrial bioenergetic, metabolic adaptation, proteostasis, inflammatory signaling, and cellular stress responses. Consequently, disruption of redox homeostasis simultaneously affects multiple interconnected functional modules, promoting progressive network destabilization rather than isolated molecular dysfunction [14,16].

Representative experimental studies demonstrate that curcumin modulates multiple components of the redox regulatory architecture, including activation of Nrf2, enhancement of glutathione-dependent antioxidant systems, preservation of thioredoxin and peroxiredoxin pathways, attenuation of carbonyl stress, and suppression of NF-κB-mediated inflammatory signaling [81]. These coordinated actions illustrate how simultaneous modulation of multiple adaptive regulatory modules can preserve physiological redox signaling and reinforce mitochondrial network resilience.

Importantly, preservation of redox homeostasis functions both as a consequence and as a determinant of mitochondrial network stability. Improved mitochondrial function reduces ROS generation, whereas maintenance of adaptive redox signaling protects mitochondrial proteins, regulatory pathways, and quality-control systems from oxidative injury. This reciprocal relationship establishes a positive adaptive feedback loop that promotes mitochondrial resilience during chronic metabolic stress [100].

Within the Mitochondrial Homeostasis Hypothesis proposed in this review, stabilization of redox network integrity emerges as a central determinant of mitochondrial adaptation. Accordingly, the biological effects of curcumin should be interpreted as representative evidence supporting a broader systems-level principle: preservation of tissue function is more likely to result from coordinated stabilization of interconnected redox regulatory networks than from selective modulation of individual molecular targets.

### 7.10. Redox Regulation Supports the Mitochondrial Homeostasis Hypothesis

The integration of proteomic, biochemical and physiological evidence positions redox regulation as a central functional module within MCBNs [46]. From this perspective, oxidative stress emerges when adaptive regulatory mechanisms become progressively overwhelmed and mitochondrial network stability is compromised. Conversely, restoration of redox homeostasis accompanies recovery of mitochondrial function, preservation of adaptive signaling, and improvement of tissue resilience during metabolic stress.

The recurrent identification of redox-associated proteins in comparative proteomic datasets, together with growing evidence for dynamic oxidative PTMs and coordinated regulation of adaptive signaling pathways, provides convergent support for the conceptual framework proposed throughout this review [68,98,101]. Rather than representing independent observations, these complementary findings indicate that preservation of mitochondrial homeostasis depends fundamentally on maintenance of redox network integrity and on the capacity of cells to coordinate adaptive responses across multiple interconnected functional modules.

Accordingly, redox regulation should not be regarded merely as a downstream consequence of mitochondrial dysfunction but as a core organizational principle governing the behavior of MCBNs. Representative experimental evidence demonstrating coordinated modulation of antioxidant defenses, inflammatory signaling, carbonyl stress, mitochondrial bioenergetics, and adaptive regulatory pathways following curcumin treatment further illustrates this systems-level principle without implying a compound-specific mechanism of action [81].

Collectively, the evidence reviewed in this section establishes the redox component of the Mitochondrial Homeostasis Hypothesis. Within this framework, mitochondrial homeostasis is interpreted as a dynamic systems-level property that emerges from coordinated regulation of bioenergetic, metabolic, redox, proteostatic, inflammatory, and signaling networks rather than from preservation of any individual mitochondrial process. This systems-level interpretation provides the conceptual foundation for the final formulation of the Mitochondrial Homeostasis Hypothesis presented in the following section.

## 8. Systems Biology Integration and the Mitochondrial Homeostasis Hypothesis

### 8.1. The Need for an Integrative Framework

Metabolic diseases such as obesity and T2D are characterized by the simultaneous disruption of multiple interconnected biological processes, including mitochondrial bioenergetics, substrate utilization, redox regulation, inflammatory signaling, proteostasis, and tissue remodeling [3,4,10]. Although each of these pathological components has been extensively investigated, their complex interactions remain incompletely understood, limiting our ability to explain how chronic metabolic stress drives progressive organ dysfunction.

Historically, these biological processes have largely been investigated as independent mechanisms, generating valuable molecular insights but often resulting in fragmented interpretations of disease progression. Contemporary systems biology and network medicine have challenged this reductionist perspective by proposing that complex diseases emerge from coordinated disturbances affecting interconnected biological networks rather than isolated molecular defects [12,19,32]. This conceptual shift provides an opportunity to reinterpret mitochondrial dysfunction within a broader organizational framework capable of integrating molecular, functional, and physiological observations.

Mitochondria occupy a uniquely strategic position within this framework because they integrate energy production, nutrient sensing, FAO, redox signaling, protein quality control, and adaptive stress responses [7,8]. Rather than functioning solely as bioenergetic organelles, mitochondria coordinate multiple regulatory processes that collectively determine cellular adaptation and tissue resilience under changing metabolic conditions. Their central position within cellular physiology therefore makes them a logical organizational hub for systems-level models of metabolic disease.

The evidence reviewed throughout this article progressively connects comparative proteomic remodeling with functional alterations, regulatory network dynamics, and physiological adaptation, revealing a consistent pattern of coordinated mitochondrial network behavior across multiple experimental models and organs. Collectively, these observations indicate that an integrative systems-level framework is required to unify these complementary lines of evidence into a coherent biological model.

### 8.2. Redefining Mitochondrial Homeostasis as an Emergent Systems-Level Property

Mitochondrial homeostasis has traditionally been interpreted as the maintenance of mitochondrial integrity through preservation of ATP production, respiratory capacity, mitochondrial abundance, membrane potential, and organelle quality control [7,23,35]. Although these parameters remain fundamental indicators of mitochondrial function, they primarily describe individual mitochondrial properties and do not fully explain how mitochondria sustain cellular adaptation during persistent metabolic stress.

Accumulating evidence indicates that mitochondrial adaptation depends on coordinated regulation of multiple interconnected biological processes rather than on preservation of any single mitochondrial function. Comparative proteomics consistently identifies conserved functional modules undergoing coordinated remodeling, while functional, biochemical, and regulatory studies demonstrate that these modules operate through continuous reciprocal interactions. This interpretation is fully consistent with systems biology, in which emergent biological properties arise from coordinated interactions among interconnected network modules rather than from the behavior of individual molecular components [12,32].

Within this framework, mitochondrial homeostasis may be redefined as the emergent systems-level property arising from coordinated regulation of MCBNs governing bioenergetics, substrate utilization, redox resilience, proteostasis, inflammatory regulation, and adaptive signaling. Importantly, these functional modules continuously exchange information through dynamic feedback mechanisms that preserve mitochondrial adaptability, cellular resilience, and tissue function despite changing metabolic demands.

This systems-level interpretation integrates the complementary organizational layers developed throughout this review. Comparative proteomics identifies conserved mitochondrial functional modules undergoing coordinated remodeling; redox proteomics reveals the dynamic post-translational regulation of these modules; functional analyses establish their physiological consequences; and regulatory networks coordinate their collective adaptive behavior. Mitochondrial homeostasis therefore emerges not as a static mitochondrial attribute but as the dynamic outcome of continuous interactions among these interconnected biological networks. This conceptual framework provides the biological foundation for interpreting metabolic disease progression and for formulating the Mitochondrial Homeostasis Hypothesis presented later in this section [46].

### 8.3. A Systems Biology Interpretation of Metabolic Disease

Within this framework, metabolic homeostasis depends on the coordinated behavior of MCBNs that continuously integrate nutrient availability, energetic demand, redox signaling, proteostasis, and adaptive regulatory pathways. Under physiological conditions, this dynamic network organization preserves mitochondrial function, metabolic flexibility, and tissue resilience by maintaining balanced communication among interconnected functional modules [43,44].

Chronic exposure to caloric excess progressively disrupts this coordinated organization. Increased substrate overload, impaired FAO, excessive ROS generation, defective proteostasis, persistent inflammatory activation, and progressive extracellular matrix remodeling collectively exceed the adaptive capacity of MCBNs [4,10,39]. Rather than representing independent pathological events, these alterations emerge through reciprocal interactions that progressively compromise coordinated mitochondrial regulation.

Accordingly, metabolic disease may be interpreted as a transition from an adaptive network state to a maladaptive network state characterized by progressive loss of coordinated mitochondrial behavior. Within this systems-level perspective, bioenergetic dysfunction, oxidative imbalance, inflammation, and fibrosis represent interconnected manifestations of a common biological process rather than independent pathogenic mechanisms. This interpretation provides a unified explanation for the remarkable convergence of molecular, functional, and physiological alterations consistently observed across experimental models of obesity and T2D [3,64].

Importantly, this conceptual framework differs from traditional models in which mitochondrial dysfunction is viewed primarily as a downstream consequence of metabolic disease. Instead, mitochondria are interpreted as central organizational hubs that integrate multiple adaptive and maladaptive regulatory processes governing tissue function [8,11,35]. Consequently, disease progression reflects progressive destabilization of coordinated mitochondrial network behavior rather than isolated impairment of individual pathways, thereby providing the systems-level context for the complementary proteomic, functional, and regulatory evidence presented in the following sections.

### 8.4. Integration of Proteomic Evidence

One of the strongest experimental foundations supporting the MCBNs framework derives from the remarkable convergence of comparative proteomic findings across tissues, experimental models, and independent investigations. Despite substantial physiological differences among the liver, heart, kidney, pancreas, and brain, proteomic analyses consistently identify coordinated remodeling affecting the same core mitochondrial functional modules, including OXPHOS, FAO, redox regulation, mitochondrial proteostasis, and adaptive stress responses [13,18,22,23].

The recurrence of these conserved functional modules across biologically distinct systems suggests that metabolic disease progression is characterized by systematic remodeling of mitochondrial network organization rather than isolated alterations in individual proteins. Importantly, this convergence extends beyond experimental reproducibility. Instead, it indicates the existence of a conserved mitochondrial functional architecture that is progressively remodeled under chronic metabolic stress, regardless of tissue-specific physiology.

Within the systems-level framework, comparative proteomics identifies the conserved mitochondrial functional modules that constitute the structural foundation of MCBNs. The reproducibility of these coordinated proteomic signatures across independent datasets should consequently be interpreted as evidence of conserved biological network architecture rather than repeated observations of individual protein dysregulation. This interpretation provides one of the principal experimental pillars supporting the MCBNs framework developed throughout this review.

Representative intervention studies further reinforce this interpretation. Multiple mitochondrial functional modules that undergo coordinated remodeling during metabolic disease exhibit partial or complete normalization following curcumin treatment [81]. Rather than implying a compound-specific mechanism, these observations illustrate the broader principle that simultaneous modulation of interconnected mitochondrial functional modules is biologically achievable.

### 8.5. Functional and Regulatory Evidence Explains Mitochondrial Network Behavior

The proteomic remodeling described is consistently accompanied by measurable functional alterations that reinforce the systems-level interpretation of MCBNs. Across experimental models of obesity and T2D, coordinated changes in mitochondrial respiration, FAO, redox regulation, proteostasis, inflammatory signaling, and tissue remodeling occur in parallel with proteomic remodeling of the corresponding functional modules [3,39,77]. This close correspondence indicates that comparative proteomic findings reflect biologically meaningful network adaptation rather than isolated molecular variation.

These functional changes are coordinated by an interconnected regulatory architecture involving energy-sensing, metabolic, redox-responsive, and inflammatory signaling pathways, including the AMPK-SIRT1-PGC-1α axis, PPAR signaling, Nrf2, and NF-κB [4,43,81,83,93]. Rather than acting independently, these regulatory modules continuously interact to coordinate mitochondrial metabolism, adaptive stress responses, redox resilience, and tissue homeostasis, thereby determining the dynamic behavior of MCBNs.

From a systems biology perspective, the convergence of proteomic remodeling, functional adaptation, and coordinated regulatory signaling provides compelling evidence that mitochondrial adaptation is governed by integrated network dynamics rather than by independent molecular pathways. Functional modules define the biological capabilities of the network, whereas regulatory pathways determine their coordinated activation, suppression, and adaptive flexibility in response to metabolic stress. This complementary relationship explains how molecular remodeling is translated into physiological outcomes across diverse experimental settings. Representative intervention studies further support this interpretation. Curcumin simultaneously enhances adaptive regulatory pathways, including AMPK-SIRT1-PGC-1α and Nrf2, while attenuating maladaptive inflammatory signaling mediated by NF-κB and related pathways [14,17,96].

### 8.6. Cross-Organ Conservation Supports a Unified Mitochondrial Network Architecture

One of the most compelling observations emerging from the evidence reviewed throughout this article is the remarkable conservation of MCBN remodeling across metabolically distinct organs. Although obesity and T2D produce tissue-specific clinical manifestations, comparative proteomic and functional studies consistently identify coordinated remodeling of the same mitochondrial functional modules across the liver, heart, kidney, pancreas, and brain [9,11,13,35].

This recurrent pattern suggests that tissue specificity reflects differences in physiological function and metabolic demand rather than fundamental differences in the underlying mitochondrial network architecture. In each organ, disease progression is characterized by coordinated remodeling of conserved mitochondrial functional modules, followed by corresponding functional and regulatory adaptation. The recurrence of this hierarchical pattern across diverse biological contexts provides strong evidence that MCBNs represent a conserved organizational principle rather than tissue-specific regulatory systems.

**Figure 3:**
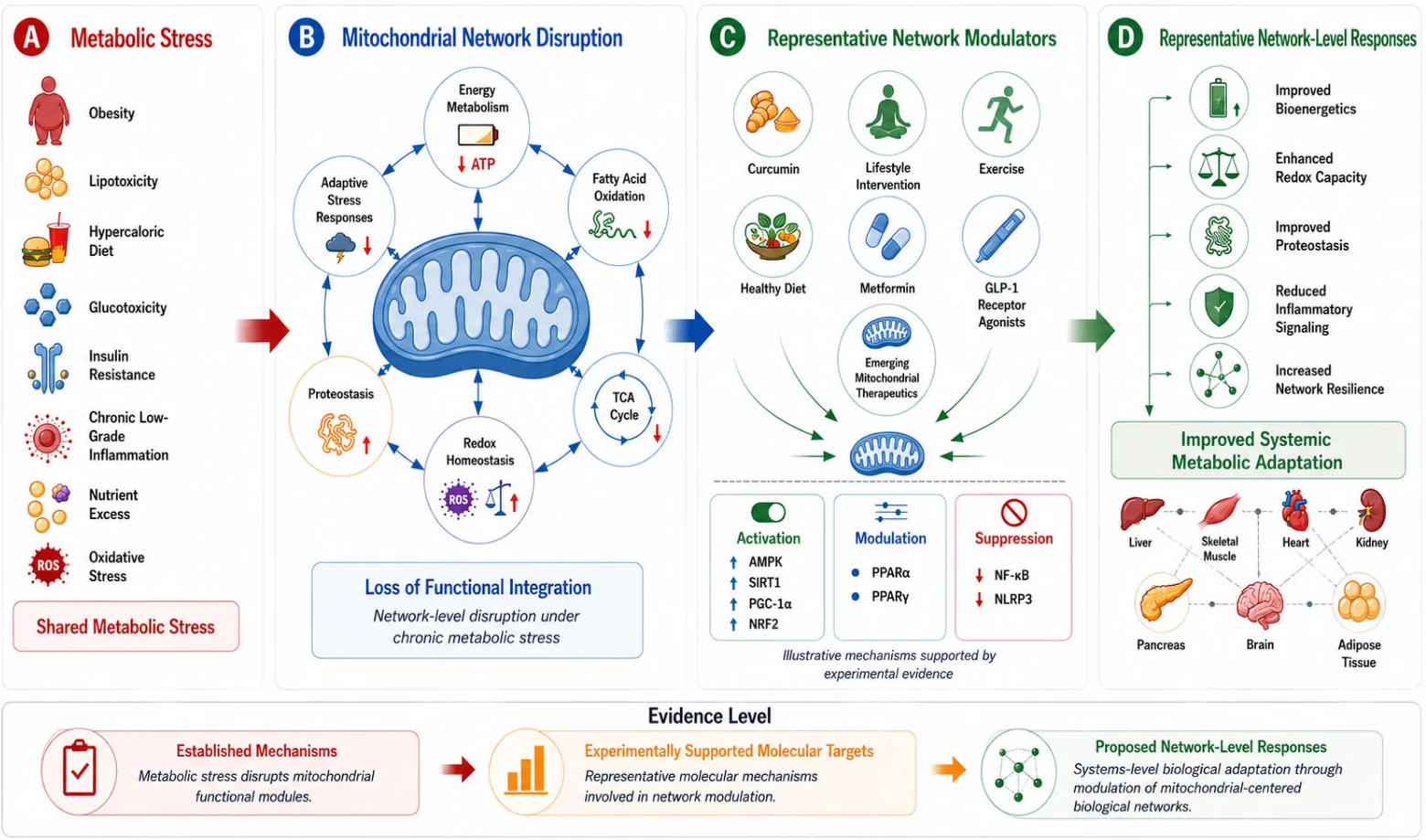
Network-Level Modulation of Mitochondrial Functional Modules Under Metabolic Stress. Chronic metabolic stress disrupts coordinated interactions among conserved mitochondrial functional modules, leading to impaired bioenergetics, oxidative imbalance, proteostatic stress, and persistent inflammatory signaling. Multiple representative interventions-including lifestyle modification, exercise, healthy diet, metformin, glucagon-like peptide-1 (GLP-1) receptor agonists, curcumin, and emerging mitochondrial therapeutics-may influence interconnected mitochondrial functional modules through complementary molecular mechanisms. Rather than acting through a single pathway, these interventions are proposed to promote systems-level improvements in mitochondrial network organization, thereby enhancing metabolic adaptation across multiple organs. (**A**) Chronic metabolic stress disrupts the coordinated interactions among conserved mitochondrial functional modules, leading to impaired bioenergetics, oxidative imbalance, proteostatic stress, and persistent inflammatory signaling. (**B**) Representative pharmacological and non-pharmacological interventions and emerging mitochondrial therapeutics may modulate interconnected mitochondrial functional modules through complementary molecular mechanisms. (**C**) Rather than acting through a single signaling pathway, these interventions are proposed to coordinately modulate multiple conserved mitochondrial functional modules, promoting restoration of network organization and functional integration. (**D**) Systems-level modulation of MCBNs enhances metabolic adaptation across multiple organs by improving mitochondrial communication, bioenergetic coordination, and cellular resilience.

This systems-level interpretation also explains why diverse therapeutic interventions, including lifestyle modification, exercise, and dietary strategies, established metabolic therapies, emerging mitochondria-directed compounds, and representative pleiotropic agents such as curcumin, can improve mitochondrial function despite acting through different primary molecular targets. Rather than converging on a single signaling pathway, these interventions appear to preserve or restore the coordinated behavior of conserved mitochondrial functional modules. Figure 3 summarizes this concept by illustrating how multiple representative interventions may stabilize mitochondrial network organization through complementary mechanisms operating at the systems level.

**Figure 4:**
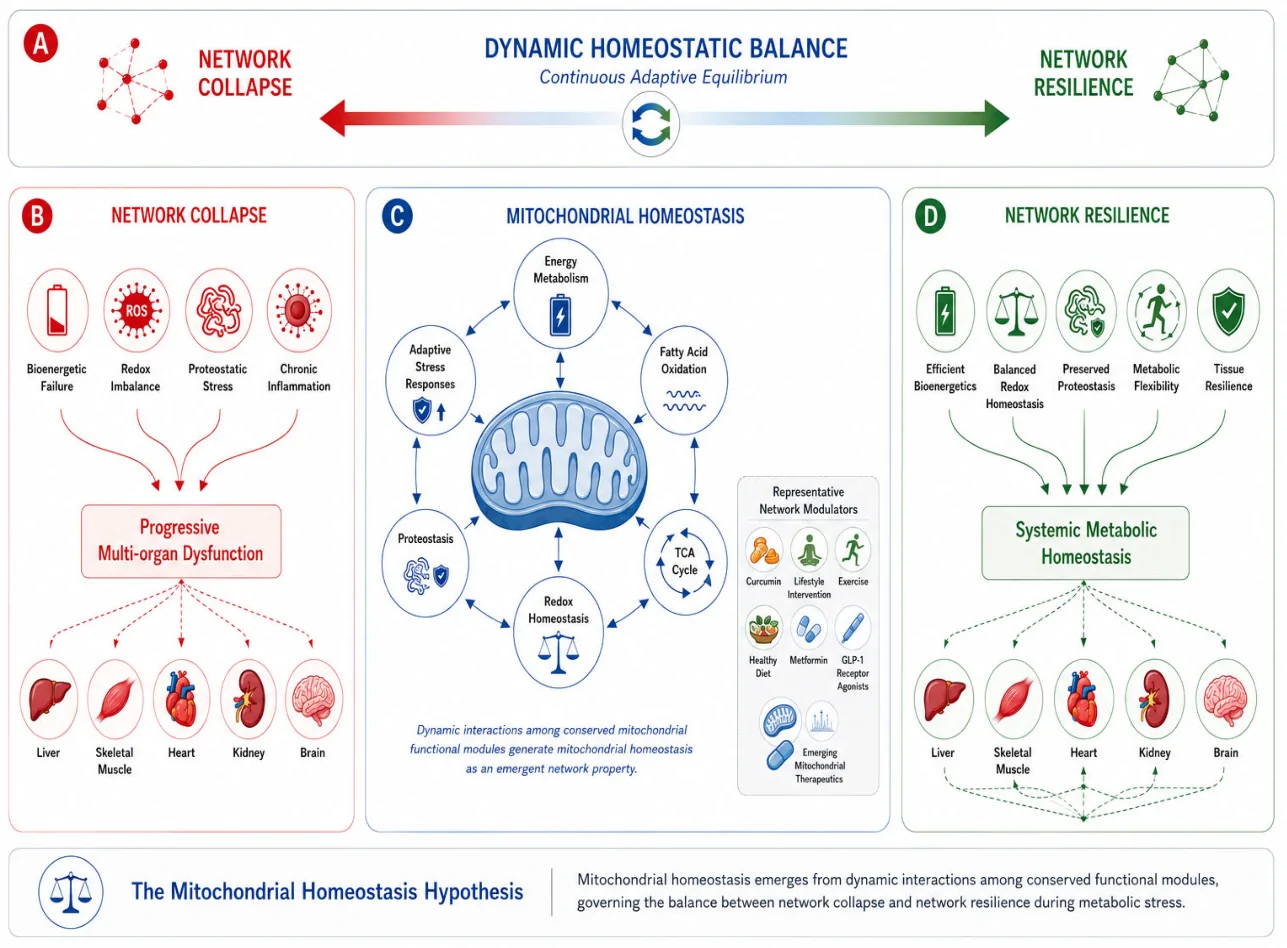
Emergence of Mitochondrial Homeostasis Through Coordinated Regulation of MCBNs. Mitochondrial homeostasis is proposed as an emergent property arising from dynamic interactions among conserved mitochondrial functional modules within MCBNs. Under sustained metabolic stress, disruption of functional integration promotes network collapse, characterized by bioenergetic failure, redox imbalance, proteostatic stress, chronic inflammation, and progressive multi-organ dysfunction. Conversely, coordinated communication among conserved functional modules enhances network resilience, supporting efficient energy production, balanced redox regulation, preserved proteostasis, metabolic flexibility, and tissue adaptation. Representative pharmacological and non-pharmacological interventions may influence this dynamic equilibrium, although the proposed hypothesis is independent of any individual therapeutic strategy. (**A**) Chronic metabolic stress disrupts the coordinated interactions among conserved mitochondrial functional modules, leading to impaired bioenergetics, oxidative imbalance, proteostatic stress, and persistent inflammatory signaling. (**B**) Representative pharmacological and non-pharmacological interventions and emerging mitochondrial therapeutics may modulate interconnected mitochondrial functional modules through complementary molecular mechanisms. (**C**) Rather than acting through a single signaling pathway, these interventions are proposed to coordinately modulate multiple conserved mitochondrial functional modules, promoting restoration of network organization and functional integration. (**D**) Systems-level modulation of MCBNs enhances metabolic adaptation across multiple organs by improving mitochondrial communication, bioenergetic coordination, and cellular resilience.

Collectively, cross-organ conservation provides one of the strongest biological arguments supporting the proposed MCBNs framework. Table 4 summarizes representative regulatory pathways that contribute to preservation of mitochondrial network organization, whereas Figure 4 integrates the experimental evidence reviewed throughout this article into the Mitochondrial Homeostasis Hypothesis. Together, these conceptual models emphasize that mitochondrial adaptation depends primarily on preservation of coordinated network behavior rather than on modulation of individual molecular pathways.

### 8.7. The Mitochondrial Homeostasis Hypothesis

The cumulative proteomic, molecular, bioenergetic, functional, signaling, and physiological evidence reviewed throughout this manuscript converges toward a unified systems-level interpretation of mitochondrial adaptation during metabolic disease. Rather than representing independent observations, these complementary findings consistently support the existence of conserved mitochondrial functional modules that undergo coordinated remodeling across tissues, experimental models, and methodological approaches [3,13,83].

Based on this integrated evidence, we propose the Mitochondrial Homeostasis Hypothesis. We hypothesize that mitochondrial homeostasis is not determined by preservation of individual mitochondrial functions but emerges from the continuous coordinated regulation of MCBNs. Within this framework, preservation of coordinated network organizations promotes mitochondrial adaptability, enhances tissue resilience, and ultimately determines the progression or attenuation of metabolic disease. Figure 4 summarizes this conceptual model by illustrating how coordinated interactions among conserved mitochondrial functional modules give rise to mitochondrial homeostasis as an emergent systems-level property.

This hypothesis integrates evidence obtained from multiple organs, complementary experimental models, and diverse methodological approaches. Comparative proteomics consistently identifies conserved mitochondrial functional modules, functional studies demonstrate the physiological consequences of their coordinated remodeling, and regulatory analyses reveal the signaling architecture governing their adaptive behavior. Together, these complementary lines of evidence support the interpretation that mitochondrial homeostasis emerges from dynamic interactions among interconnected biological networks rather than from the activity of individual molecular pathways [3,13,83].

Importantly, the proposed hypothesis does not assign a dominant role to any single molecular pathway or therapeutic intervention. Instead, it postulates that preservation of coordinated mitochondrial network integrity constitutes the fundamental biological principle linking molecular remodeling with functional adaptation and physiological outcomes [12]. Under sustained metabolic stress, progressive disruption of communication among conserved mitochondrial functional modules shifts the network toward maladaptive states characterized by bioenergetic dysfunction, redox imbalance, proteostatic stress, chronic inflammation, and tissue injury. Conversely, preservation or restoration of coordinated network behavior promotes mitochondrial resilience, metabolic flexibility, and tissue adaptation, thereby establishing the conceptual foundation for the experimental predictions presented in the following section.

### 8.8. Implications and Experimental Predictions

The Mitochondrial Homeostasis Hypothesis generates a series of experimentally testable predictions that can guide future investigation of mitochondrial adaptation during metabolic disease. First, MCBNs should consistently emerge as conserved regulatory modules across additional tissues, disease models, and independent proteomic datasets, despite differences in organ-specific physiology. Second, coordinated remodeling of mitochondrial functional modules should correlate more closely with functional outcomes than alterations in individual mitochondrial proteins or isolated signaling pathways. Third, restoration of coordinated network organization should consistently precede or accompany improvements in mitochondrial function, metabolic flexibility, redox homeostasis, proteostasis, inflammatory regulation, and tissue remodeling, reflecting recovery of integrated biological network behavior rather than isolated molecular correction [13,34].

These predictions provide a clear experimental framework for evaluating the proposed hypothesis. Comparative quantitative proteomics, redox proteomics, spatial proteomics, acetylomics, single-cell proteomics, multi-omics integration, and artificial intelligence-assisted systems biology offer complementary approaches for characterizing mitochondrial network organization across multiple biological scales [37,48,49,111]. These methodologies collectively enable reconstruction of the structural, functional, regulatory, and post-translational architecture of MCBNs, thereby allowing direct evaluation of the Mitochondrial Homeostasis Hypothesis. Validation of this framework would also have important translational implications.

Network-level biomarkers derived from coordinated remodeling of conserved mitochondrial functional modules may provide more robust indicators of disease progression and therapeutic responsiveness than individual molecular markers. Likewise, interventions that preserve or restore coordinated mitochondrial network behavior are predicted to produce broader and more durable physiological benefits than strategies directed toward single molecular targets [34,35]. Importantly, this prediction is independent of any individual pharmacological or non-pharmacological intervention and reflects a general principle of systems-level mitochondrial regulation.

Collectively, these experimental predictions establish a practical research agenda for testing and refining the proposed framework. Confirmation of the Mitochondrial Homeostasis Hypothesis would support a transition from reductionist models of mitochondrial dysfunction toward a network-oriented understanding of metabolic disease, providing a robust conceptual foundation for future studies and for the development of precision mitochondrial medicine based on preservation of coordinated mitochondrial network organization.

## 9. Future Perspectives and Emerging Technologies

### 9.1. Moving Beyond Conventional Proteomics

Comparative proteomics has substantially expanded our understanding of obesity, T2D, and hypercaloric diet-induced metabolic dysfunction by revealing coordinated remodeling of MCBNs. As discussed throughout this review, quantitative proteomic analyses have consistently identified conserved mitochondrial functional modules undergoing coordinated remodeling across multiple organs and experimental models, thereby providing the principal experimental foundation for the proposed MCBNs framework [13,18]. Nevertheless, most available studies primarily quantify changes in protein abundance, providing only a partial representation of mitochondrial adaptation during metabolic stress.

Mitochondrial function is determined not only by protein abundance but also by PTMs, protein–protein interactions, subcellular localization, supramolecular organization, and dynamic regulatory signaling [101]. Consequently, quantitative proteomic data alone cannot fully explain how mitochondrial functional modules interact, adapt, and maintain coordinated network behavior during health and disease. Understanding mitochondrial homeostasis therefore requires approaches capable of integrating both the structural composition and the functional regulation of MCBNs.

Accordingly, the next generation of proteomic research should move beyond descriptive cataloging of mitochondrial proteins toward integrated characterization of mitochondrial network architecture and regulatory dynamics. Functional proteomics and complementary high-resolution technologies will enable reconstruction of MCBNs across multiple organizational levels, providing the experimental framework necessary to evaluate the Mitochondrial Homeostasis Hypothesis proposed in this review. The following sections discuss how these emerging technologies contribute complementary information for understanding the structural, functional, spatial, and regulatory organization of MCBNs.

### 9.2. Redox Proteomics and Functional Characterization of Mitochondrial Adaptation

Among the most important advances in functional proteomics is the emergence of redox proteomics, which extends conventional protein abundance analysis by characterizing oxidative PTMs that directly regulate protein function. Unlike quantitative proteomics alone, redox proteomics identifies the specific molecular events that influence enzyme activity, protein stability, protein–protein interactions, and adaptive signaling, thereby providing mechanistic insight into the functional state of the mitochondrial proteome [60,68,105]. This additional functional dimension is particularly valuable for understanding how MCBNs respond dynamically to metabolic stress.

Oxidative PTMs-including protein carbonylation, S-nitrosylation, S-glutathionylation, and cysteine sulfenylation-are increasingly recognized as reversible regulatory mechanisms rather than merely indicators of oxidative damage [98,99]. These modifications modulate the activity and coordination of proteins participating in OXPHOS, FAO, mitochondrial proteostasis, antioxidant defense, and adaptive signaling. Consequently, redox proteomics provides direct insight into the regulatory architecture that coordinates communication among conserved mitochondrial functional modules and contributes to the maintenance of mitochondrial homeostasis.

Integration of redox proteomics with quantitative proteomic analyses offers an opportunity to distinguish adaptive redox signaling from persistent oxidative injury while simultaneously identifying regulatory nodes that govern mitochondrial network behavior during metabolic stress [46]. Ultimately, redox proteomics may provide one of the most direct experimental approaches for validating the Mitochondrial Homeostasis Hypothesis proposed in this review.

### 9.3. Spatial Proteomics and Tissue Microenvironment Heterogeneity

A major limitation of conventional proteomic analyses is the loss of spatial information during tissue homogenization, which obscures cellular organization and prevents precise localization of molecular alterations. Emerging spatial proteomic technologies overcome this limitation by enabling comprehensive protein characterization while preserving tissue architecture and cellular context [112,113]. Consequently, spatial proteomics provides an unprecedented opportunity to investigate MCBNs within their native anatomical environment rather than as averaged molecular profiles derived from heterogeneous tissues.

This spatial dimension is particularly important in metabolic diseases, where mitochondrial dysfunction frequently develops within anatomically and functionally distinct tissue microenvironments. Regional differences in metabolic demand, oxygen availability, inflammatory activity, and cellular composition generate localized patterns of mitochondrial remodeling that cannot be resolved by conventional bulk proteomic analyses. Spatial proteomics therefore enables reconstruction of MCBNs within discrete anatomical compartments, revealing how local alterations in mitochondrial network organization contribute to tissue dysfunction and disease progression.

By preserving anatomical context, spatial proteomics expands the study of mitochondrial adaptation beyond protein abundance and functional regulation to include the organizational architecture of biological networks across tissues. Integration of spatial proteomics with quantitative proteomics, redox proteomics, and complementary multi-omics approaches will facilitate multidimensional reconstruction of mitochondrial network behavior across different organs and stages of metabolic disease. Moreover, characterization of tissue microenvironments provides the conceptual bridge toward single-cell proteomics, where MCBNs can be investigated at the resolution of individual cells while maintaining their relationship to the surrounding tissue architecture.

### 9.4. Single-Cell Proteomics and Cellular Resolution of MCBNs

Single-cell proteomics represents one of the most rapidly evolving technologies in biomedical research and has the potential to transform our understanding of mitochondrial biology by enabling comprehensive protein characterization at the resolution of individual cells [110,111]. Unlike conventional bulk tissue analyses, which average molecular information across heterogeneous cellular populations, single-cell proteomics reveals the diversity of MCBNs operating within distinct cell types while preserving the complexity of multicellular tissues [114,115]. This unprecedented level of resolution provides new opportunities to investigate how individual cellular states collectively shape mitochondrial adaptation during metabolic disease.

Within metabolically active organs, individual cell populations differ substantially in their energetic requirements, metabolic programs, inflammatory responses, and susceptibility to injury. Consequently, mitochondrial adaptation is unlikely to occur uniformly throughout a tissue but instead reflects coordinated yet heterogeneous remodeling of cell-specific MCBNs. Single-cell proteomics enables identification of distinct cellular trajectories associated with adaptation, compensation, dysfunction, and tissue remodeling, thereby providing a more accurate representation of mitochondrial network behavior during obesity, T2D, and hypercaloric diet-induced metabolic stress. Representative therapeutic interventions, including curcumin, may serve as useful experimental models for exploring these cell-specific responses without constituting the primary focus of this technology.

By linking molecular remodeling within individual cells to tissue-level physiological behavior, single-cell proteomics provides a critical bridge between cellular heterogeneity and the emergent properties that define mitochondrial homeostasis. Integration of single-cell proteomics with spatial proteomics, quantitative proteomics, redox proteomics, transcriptomics, metabolomics, and other complementary multi-omics approaches will enable increasingly comprehensive reconstruction of MCBNs across multiple biological scales [116]. These integrative analyses are expected to clarify how coordinated interactions among diverse cellular populations generate tissue resilience or dysfunction, while also preparing the framework for investigating the post-translational regulatory mechanisms that further modulate mitochondrial network behavior.

### 9.5. Acetylomics and Post-Translational Regulation of Mitochondrial Function

Accumulating evidence indicates that mitochondrial metabolism is extensively regulated by lysine acetylation and other PTMs, which dynamically modulate the activity of proteins involved in OXPHOS, the TCA cycle, FAO, and numerous additional metabolic pathways [101,117,118]. Within this regulatory framework, NAD^+^-dependent deacetylases such as SIRT1 occupy central positions by coupling cellular energetic status to reversible post-translational control of mitochondrial proteins [44,45,108]. Consequently, acetylomic analyses offer an opportunity to characterize how post-translational regulation contributes to the coordinated behavior of conserved mitochondrial functional modules under physiological and pathological conditions.

Future investigations should therefore consider mitochondrial proteome remodeling as a multidimensional process involving coordinated changes in protein abundance together with multiple layers of post-translational regulation. Integration of acetylomics with quantitative proteomics, redox proteomics, spatial proteomics, single-cell proteomics, and other PTM-focused approaches will provide a more comprehensive understanding of the regulatory mechanisms governing mitochondrial adaptation. By combining these complementary datasets, researchers will be able to reconstruct both the structural organization and the dynamic regulatory architecture of MCBNs, thereby establishing the foundation for systems-level analyses.

### 9.6. Multi-Omics Integration and Reconstruction of MCBNs

Perhaps the most important future direction in mitochondrial research is the integration of complementary omics platforms into unified systems-level models. Transcriptomics, proteomics, metabolomics, lipidomics, and epigenomics each provide distinct yet complementary perspectives on cellular adaptation during metabolic disease [48,49]. Although each technology offers valuable biological information independently, none can fully capture the complexity of MCBNs. Multi-omics integration therefore represents a fundamental shift from analyzing isolated molecular layers toward understanding coordinated biological systems.

From a systems biology perspective, multi-omics approaches are uniquely suited to reconstruct MCBNs because they simultaneously capture molecular remodeling occurring at transcriptional, translational, post-translational, metabolic, and epigenetic levels. Integration of these complementary datasets enables dynamic regulatory networks to be reconstructed rather than simply cataloging individual molecular pathways. Consequently, multi-omics analyses provide a more comprehensive representation of mitochondrial adaptation by linking molecular regulation with functional behavior across multiple biological scales.

Artificial intelligence-assisted network reconstruction, machine learning, and advanced computational systems biology are expected to play an increasingly important role in integrating these highly complex datasets [34,37]. Rather than functioning as independent analytical tools, these computational approaches facilitate identification of highly connected regulatory hubs, prediction of network behavior, and characterization of emergent interactions among bioenergetic, metabolic, redox, proteostatic, and signaling modules. Such integrative modeling is essential for transforming multidimensional molecular information into biologically interpretable MCBNs.

Accordingly, future research should move beyond the parallel analysis of individual omics datasets toward quantitative reconstruction of dynamic MCBNs capable of explaining mitochondrial adaptation across tissues, disease stages, and therapeutic interventions. Table 5 summarizes how emerging omics technologies contribute complementary information across successive organizational levels, from protein abundance and post-translational regulation to cellular heterogeneity, tissue architecture, and systems-level network behavior. Collectively, these integrated approaches provide the experimental and computational framework required to investigate mitochondrial homeostasis as an emergent property of coordinated biological networks, establishing the methodological foundation for the development of precision mitochondrial medicine.

### 9.7. Emerging Technologies Support the Development of Precision Mitochondrial Medicine

Collectively, the emerging technologies discussed throughout this section are transforming mitochondrial research from a predominantly descriptive discipline into a predictive, mechanistic, and systems-level science. Quantitative proteomics, redox proteomics, spatial proteomics, single-cell proteomics, acetylomics, multi-omics integration, and artificial intelligence-assisted network analysis each contribute complementary layers of biological information that progressively increase the resolution at which MCBNs can be reconstructed. Rather than generating isolated molecular datasets, these integrated approaches enable comprehensive characterization of the structural, functional, regulatory, spatial, and cellular architecture underlying mitochondrial homeostasis [34,37].

Beyond advancing mechanistic understanding, reconstruction of MCBNs has important translational implications. Network-level biomarkers derived from coordinated remodeling of conserved mitochondrial functional modules may provide more accurate indicators of mitochondrial homeostasis, tissue resilience, disease progression, and therapeutic responsiveness than individual molecular markers. Likewise, systems-level characterization of mitochondrial network organization may facilitate patient stratification, identification of biologically relevant disease phenotypes, and development of therapeutic strategies aimed at preserving coordinated network integrity rather than targeting isolated molecular pathways [36].

Ultimately, the convergence of advanced proteomics, complementary multi-omics technologies, systems biology, and computational network analysis provides the experimental framework required to investigate mitochondrial homeostasis as a dynamic and quantifiable systems-level property. These integrated methodologies create the opportunity to translate the Mitochondrial Homeostasis Hypothesis from a conceptual model into an experimentally testable and clinically applicable framework. More broadly, they establish the scientific foundation for precision mitochondrial medicine, in which preservation or restoration of MCBN integrity becomes a principal objective for preventing or attenuating metabolic disease.

## 10. Toward Precision Mitochondrial Medicine

### 10.1. From Mitochondrial Biology to Precision Medicine

The growing recognition of mitochondria as central regulators of metabolic adaptation, together with recent advances in proteomics, multi-omics integration, systems biology, and computational network analysis, has created new opportunities to translate fundamental mitochondrial biology into clinical practice. Current therapeutic strategies for obesity and T2D primarily target downstream manifestations of disease, including hyperglycemia, dyslipidemia, hypertension, and inflammation, have produced substantial clinical benefits. Nevertheless, these approaches frequently fail to address the underlying network-level mechanisms responsible for progressive mitochondrial dysfunction and loss of tissue adaptability [3,4,35].

The Mitochondrial Homeostasis Hypothesis proposed in this review offers an alternative conceptual framework for therapeutic intervention. Rather than focusing exclusively on isolated molecular targets or individual pathological endpoints, future therapeutic strategies may seek to preserve or restore the coordinated organization of MCBNs. Because these interconnected networks collectively regulate bioenergetics, substrate utilization, redox homeostasis, mitochondrial proteostasis, inflammatory signaling, and adaptive responses, maintenance of mitochondrial homeostasis is predicted to promote broader and more durable physiological adaptation than interventions directed toward single molecular pathways.

Within this systems-oriented perspective, therapeutic efficacy depends not only on correcting metabolic abnormalities but also on restoring coordinated mitochondrial network behavior. This conceptual shift closely aligns with the objectives of precision medicine, which seeks to characterize biological heterogeneity among patients and tailor therapeutic strategies according to their underlying molecular and functional profiles [34,48,119]. Within this emerging framework, mitochondrial homeostasis becomes a measurable systems-level phenotype that integrates molecular regulation, network behavior, and tissue function. Consequently, future precision mitochondrial medicine will require biomarkers capable of quantitatively assessing the organization and adaptive capacity of MCBNs rather than relying exclusively on conventional metabolic indicators.

### 10.2. Biomarkers of Mitochondrial Homeostasis

A critical step toward precision mitochondrial medicine is the development of biomarkers capable of quantitatively assessing mitochondrial homeostasis and the functional integrity of MCBNs. Conventional metabolic biomarkers, including glucose, glycated hemoglobin, triglycerides, and cholesterol, remain indispensable for clinical management but primarily reflect systemic metabolic status rather than the adaptive capacity of mitochondrial networks. Advances in proteomics and complementary omics technologies now provide the opportunity to identify molecular signatures that more directly capture the functional state of mitochondrial adaptation and its progressive remodeling during metabolic disease.

Rather than relying on individual molecules, future biomarkers are likely to consist of coordinated molecular signatures representing conserved mitochondrial functional modules, OXPHOS, FAO, antioxidant defense, mitochondrial proteostasis, adaptive signaling pathways, and redox homeostasis. Because these biological processes operate as highly interconnected components of MCBNs, their coordinated behavior is expected to provide a more comprehensive assessment of mitochondrial homeostasis than isolated biochemical measurements. Accordingly, the concept of network-based biomarkers emerges as a logical extension of the Mitochondrial Homeostasis Hypothesis, emphasizing preservation of coordinated network organization rather than changes in individual molecular targets.

Within this framework, mitochondrial homeostasis becomes a measurable systems-level phenotype defined by integrated molecular signatures rather than by single biochemical parameters. The multidimensional nature of mitochondrial adaptation suggests that no individual protein or metabolite is likely to provide sufficient diagnostic accuracy. Instead, quantitative assessment of coordinated molecular modules and their regulatory interactions will require integration of proteomic, functional proteomic, transcriptomic, metabolomic, lipidomic, and computational network analyses [13,34,37,49].

### 10.3. Multi-Omics Signatures of Mitochondrial-Centered Biological Networks

The integration of transcriptomic, proteomic, metabolomic, lipidomic, and epigenomic information provides an unprecedented opportunity to define comprehensive molecular signatures of mitochondrial homeostasis. Rather than describing isolated molecular alterations, multi-omics approaches characterize coordinated biological responses across multiple regulatory levels, thereby generating systems-level representations of mitochondrial adaptation during metabolic disease [48]. These integrated molecular signatures provide a more complete description of MCBNs than any individual omics platform alone.

Within this framework, each omics layer contributes complementary biological information. Transcriptomics reflects adaptive gene expression programs associated with mitochondrial biogenesis and stress responses; proteomics characterizes conserved functional modules involved in OXPHOS, FAO, redox homeostasis, and mitochondrial proteostasis; metabolomics captures substrate utilization and bioenergetic remodeling; lipidomics provides insight into metabolic flexibility and lipotoxicity; and epigenomics reveals longer-term regulatory adaptations. When interpreted collectively rather than independently, these complementary datasets generate multidimensional molecular signatures that more accurately reflect the functional state of MCBNs [37].

Integration of these diverse molecular datasets through systems biology and computational network analysis enables quantitative reconstruction of coordinated mitochondrial network states. Artificial intelligence and machine learning provide valuable analytical tools for identifying highly connected regulatory hubs, recognizing complex molecular patterns, and modeling dynamic interactions among conserved mitochondrial functional modules [34]. Importantly, the objective of these computational approaches is not simply data integration but the generation of biologically interpretable network signatures capable of describing mitochondrial homeostasis as a dynamic systems-level property. These integrated molecular signatures are expected to reveal biologically meaningful differences among patients that remain undetectable using conventional diagnostic approaches. Individuals with similar clinical diagnoses may exhibit distinct patterns of MCBN organization, stability, and adaptive capacity, reflecting fundamentally different mechanisms of disease progression. Consequently, multi-omics-derived network signatures provide the foundation for defining mitochondrial phenotypes based on coordinated network behavior rather than isolated molecular abnormalities.

### 10.4. Patient Stratification Based on Mitochondrial Phenotypes

One of the major limitations of current therapeutic approaches for obesity and T2D is the assumption that patients with similar clinical diagnoses constitute relatively homogeneous biological populations. Increasing evidence, however, indicates that substantial molecular and functional heterogeneity exists among individuals despite comparable clinical manifestations [49,119]. Within the framework of the Mitochondrial Homeostasis Hypothesis, these differences may reflect distinct organizational states of MCBNs, resulting in variable capacities for metabolic adaptation, tissue resilience, and disease progression.

Accordingly, mitochondrial phenotypes should be viewed as systems-level representations of coordinated network behavior rather than as isolated molecular defects. For example, different patients may exhibit predominant alterations involving OXPHOS, FAO, redox homeostasis, mitochondrial proteostasis, inflammatory signaling, or combinations of these conserved functional modules. These patterns are best interpreted as distinct modes of MCBN remodeling, each reflecting a unique balance between adaptive and maladaptive network responses rather than discrete disease categories. Recognition of these biologically distinct mitochondrial phenotypes has important implications for therapeutic decision-making. Instead of assuming that all patients with similar clinical characteristics will respond similarly to treatment, future precision mitochondrial medicine may seek to match therapeutic strategies with the predominant pattern of network dysfunction identified in each individual.

Such an approach emphasizes restoration of coordinated mitochondrial network function while acknowledging that different patients may require different interventions to achieve comparable improvements in mitochondrial homeostasis and metabolic adaptation. Future integration of proteomics, functional proteomics, multi-omics technologies, systems biology, and computational network analysis may therefore enable patient stratification based on quantitative MCBN phenotypes rather than solely on conventional clinical parameters [32,36]. This biologically informed classification has the potential to improve prediction of therapeutic responsiveness, refine disease monitoring, and support individualized clinical decision-making.

### 10.5. Mitochondria-Directed Therapeutic Strategies

The Mitochondrial Homeostasis Hypothesis has important implications for therapeutic development by reframing the objectives of intervention in metabolic disease. Conventional therapies have achieved substantial clinical benefits through the management of hyperglycemia, dyslipidemia, inflammation, and other metabolic abnormalities. However, increasing understanding of mitochondrial biology suggests that sustained clinical improvement may also depend on preserving the coordinated adaptive mechanisms that maintain mitochondrial homeostasis across multiple tissues. Within this context, future therapeutic strategies may complement existing approaches by targeting the systems-level processes that govern mitochondrial adaptation rather than focusing exclusively on individual molecular abnormalities.

Emerging therapeutic approaches increasingly seek to enhance mitochondrial resilience by promoting coordinated regulation of biological processes such as mitochondrial biogenesis, oxidative metabolism, FAO, redox homeostasis, mitochondrial proteostasis, metabolic flexibility, and quality-control mechanisms [8,61]. Importantly, these adaptive processes do not function independently but operate as highly interconnected components of MCBNs. Consequently, therapeutic efficacy is likely to depend on the extent to which interventions restore coordinated network function rather than on modulation of any single pathway or molecular target [35].

This systems-oriented perspective supports the concept of network-oriented therapeutics, in which preservation or restoration of coordinated MCBN organization becomes the principal therapeutic objective. Rather than evaluating interventions solely according to their effects on individual biomarkers or signaling pathways, future therapeutic development may increasingly assess their capacity to improve mitochondrial adaptability, reinforce tissue resilience, and stabilize the coordinated behavior of mitochondrial biological networks. Representative interventions capable of influencing multiple adaptive pathways, including curcumin and other emerging mitochondrial modulators, provide useful experimental examples of this broader therapeutic concept.

### 10.6. Curcumin as a Prototype Network Stabilizer

The evidence reviewed throughout this manuscript suggests that curcumin provides a representative experimental example of how a single intervention can influence multiple components of MCBNs. Across diverse experimental models, curcumin has consistently been associated with coordinated modulation of OXPHOS, FAO, redox homeostasis, mitochondrial proteostasis, inflammatory signaling, and adaptive metabolic responses, together with regulation of signaling pathways including AMPK, SIRT1, PGC-1α, PPARs, Nrf2, and NF-κB [14,17,96,101]. Rather than considering these observations as isolated molecular effects, they are more appropriately interpreted as evidence of coordinated network remodeling.

Within the framework of the Mitochondrial Homeostasis Hypothesis, curcumin may therefore be viewed as a prototype network stabilizer. Its biological significance derives not from modulation of any individual molecular target, but from its apparent capacity to preserve or restore coordinated interactions among conserved mitochondrial functional modules. This systems-level interpretation provides a unifying explanation for the broad spectrum of protective effects reported across multiple tissues and experimental models despite substantial differences in their physiological characteristics and pathological manifestations. Importantly, the conceptual framework proposed in this review extends well beyond curcumin itself. The broader implication is that future therapeutic agents may be evaluated according to their ability to preserve mitochondrial homeostasis through coordinated regulation of MCBNs rather than through selective modulation of individual signaling pathways. In this context, curcumin serves as a representative proof-of-concept supporting the broader principle of network-oriented therapeutics, while the underlying framework remains applicable to diverse classes of interventions capable of stabilizing mitochondrial biological networks.

### 10.7. Precision Mitochondrial Medicine: A Future Clinical Paradigm

Taken together, the evidence reviewed throughout this manuscript supports the emergence of precision mitochondrial medicine as a systems-oriented extension of contemporary precision medicine. Within this conceptual framework, mitochondrial homeostasis becomes a measurable, dynamic, and therapeutically actionable biological objective. Rather than focusing exclusively on conventional clinical manifestations such as hyperglycemia, dyslipidemia, or organ-specific complications, this approach seeks to characterize the MCBNs that govern metabolic adaptation, tissue resilience, and disease susceptibility [36].

Implementation of this framework will depend on integrating complementary molecular and computational approaches capable of quantitatively characterizing patient-specific MCBNs. Comparative mitochondrial proteomics, functional proteomics, multi-omics profiling, systems biology, and computational network analysis collectively enable reconstruction of network-level molecular signatures that capture the organizational and functional state of mitochondrial homeostasis. These integrated signatures provide the biological basis for identifying distinct mitochondrial phenotypes, improving patient stratification, and guiding individualized therapeutic strategies aimed at preserving or restoring coordinated network function [34].

**Figure 5:**
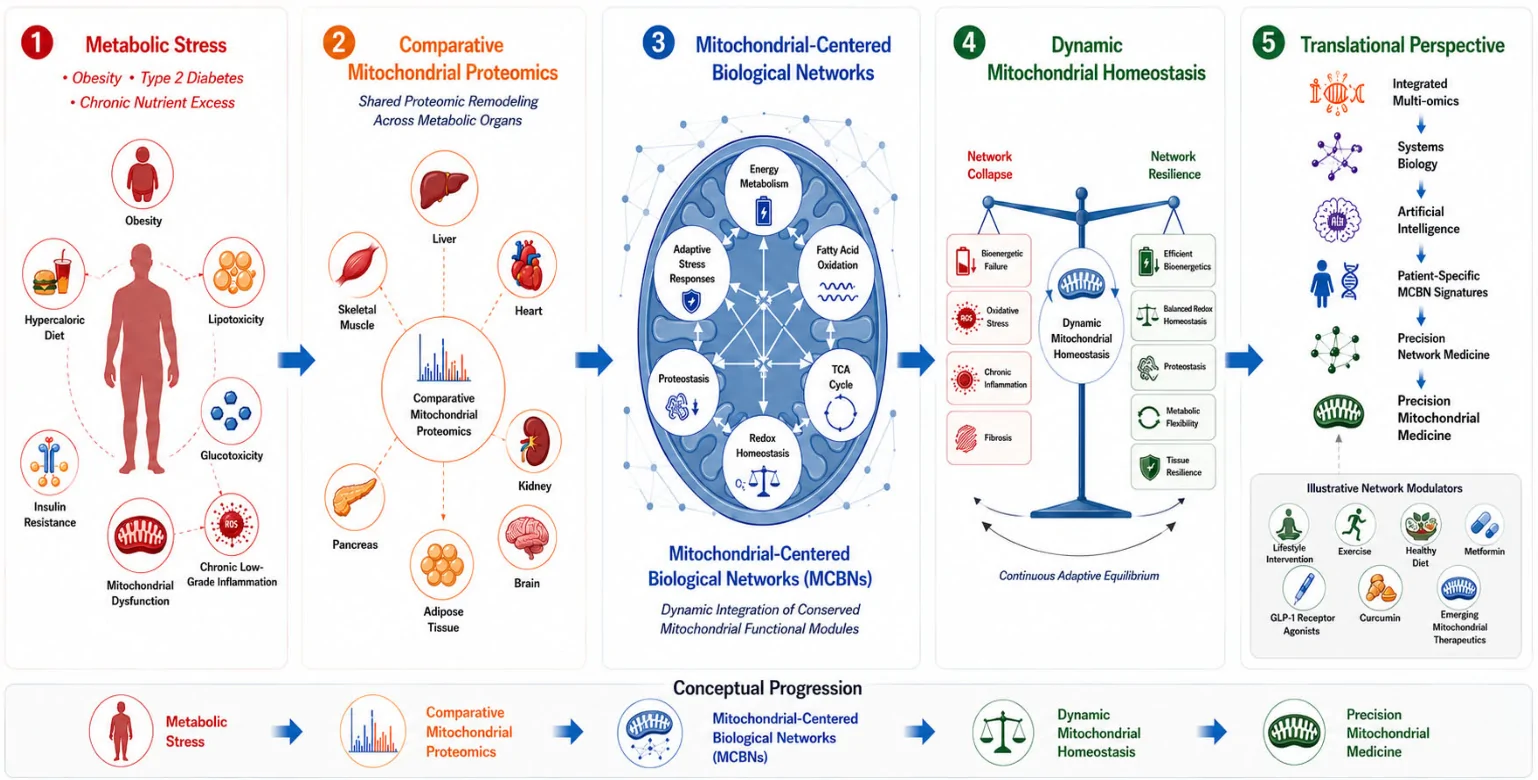
Translational Framework for Precision Mitochondrial Medicine Based on Mitochondrial-Centered Biological Networks. The proposed framework integrates comparative mitochondrial proteomics, multi-omics, systems biology, and artificial intelligence to generate patient-specific MCBN signatures. These network-level signatures provide the basis for precision network medicine by enabling the identification of individualized mechanisms of mitochondrial dysfunction and facilitating personalized therapeutic strategies. Representative network modulators-including lifestyle interventions, exercise, dietary modification, metformin, GLP-1 receptor agonists, curcumin, and emerging mitochondrial therapeutics, are presented as illustrative examples of interventions that may target MCBNs within future precision mitochondrial medicine approaches.

The proposed translational framework is summarized in Figure 5. Building on the Mitochondrial Homeostasis Hypothesis, this framework illustrates how experimental observations obtained through comparative proteomics, complementary multi-omics technologies, systems biology, and artificial intelligence-assisted network analysis converge to generate patient-specific MCBN signatures. These network-level signatures establish the foundation for precision mitochondrial medicine by linking systems-level biological characterization with individualized therapeutic decision-making and longitudinal assessment of mitochondrial homeostasis.

Although substantial experimental and clinical validation remains necessary before this framework can be fully implemented, continuing advances in proteomics, spatial and single-cell technologies, computational biology, and systems medicine are rapidly expanding the feasibility of this approach [48,111,113,120]. More broadly, the mitochondrial Homeostasis Hypothesis provides a unifying conceptual framework that integrates molecular mechanisms, mitochondrial biology, systems-level regulation, and translational medicine into a coherent model of metabolic disease. Within this perspective, preservation or restoration of coordinated MCBN organization emerges as a central therapeutic objective, offering a new foundation for the prevention, diagnosis, and treatment of obesity, T2D, and other chronic disorders characterized by mitochondrial dysfunction.

## 11. Limitations and Future Challenges

Despite the substantial advances achieved through comparative mitochondrial proteomics, functional multi-omics, and systems biology approaches, several important limitations should be considered when interpreting the evidence discussed throughout this review. As with any emerging conceptual framework, the Mitochondrial Homeostasis Hypothesis requires continued experimental evaluation across multiple biological contexts. Rather than diminishing the proposed framework, these limitations define the research priorities necessary for its validation, refinement, and eventual clinical translation. Accordingly, they should be viewed not as weaknesses of the hypothesis itself, but as essential directions for future investigation within the evolving field of precision mitochondrial medicine.

First, a substantial proportion of the evidence supporting the proposed framework derives from experimental animal models, including diet-induced obesity, diabetic rodents, and genetically modified mice. These models have provided valuable mechanistic insights into mitochondrial adaptation and the coordinated regulation of MCBNs. However, important differences in metabolic physiology, disease progression, environmental exposures, and genetic heterogeneity may limit the direct translation of these findings to human populations [3,121]. Accordingly, future investigations integrating well-characterized clinical cohorts with comparative proteomics, functional multi-omics, and longitudinal phenotypic characterization will be essential for determining whether the organizational principles underlying mitochondrial homeostasis are conserved across human metabolic diseases and can reliably support clinical translation.

Second, considerable methodological heterogeneity remains among proteomic studies investigating mitochondrial biology. Differences in experimental design, sample preparation, mass spectrometry platforms, protein quantification strategies, and bioinformatic workflows complicate direct comparisons across studies and may contribute to variability in protein identification, pathway enrichment analyses, and network reconstruction [13,18]. As the field progresses toward systems-level characterization of MCBNs, greater standardization of analytical pipelines, quality control procedures, data processing strategies, and computational approaches will be essential to improve reproducibility, facilitate cross-study integration, and enable robust reconstruction of biologically meaningful mitochondrial network signatures.

Third, most currently available proteomic studies primarily quantify changes in protein abundance and therefore provide only static molecular snapshots of mitochondrial biology. However, mitochondrial homeostasis is an intrinsically dynamic systems-level property that emerges from coordinated regulation across multiple biological layers, including PTMs, protein–protein interactions, subcellular organization, metabolic fluxes, and adaptive signaling networks. Consequently, quantitative proteomic changes alone cannot fully capture the functional state or adaptive capacity of MCBNs [117,118]. Future integration of phosphoproteomics, acetylomics, redox proteomics, spatial and single-cell proteomics, interactomics, and longitudinal multi-omics approaches will therefore be essential to characterize the dynamic organization, regulatory plasticity, and temporal behavior of mitochondrial homeostasis.

Fourth, although the Mitochondrial Homeostasis Hypothesis is supported by convergent evidence from comparative proteomics, biochemistry, physiology, and mitochondrial signaling, it should currently be regarded as a testable systems biology framework rather than a definitively established biological model. Future investigations should therefore be specifically designed to determine whether preservation or restoration of MCBN integrity consistently predicts improved physiological resilience and metabolic outcomes across diverse tissues, disease models, therapeutic interventions, and human populations. Such studies will be essential to distinguish causal network mechanisms from associative molecular changes and to establish the predictive value of mitochondrial homeostasis as a measurable systems-level biological phenotype.

Fifth, important translational questions remain regarding representative network-modulating interventions, including curcumin. Although curcumin has consistently demonstrated pleiotropic effects across multiple experimental models, its relatively low oral bioavailability, together with substantial variability among formulations, dosing regimens, and experimental protocols, complicates direct comparisons across studies and limits the interpretation of translational efficacy [15,122]. These challenges highlight the need for standardized preclinical and clinical investigations capable of linking coordinated modulation of MCBNs with clinically meaningful outcomes. Future studies should also determine whether preservation of mitochondrial homeostasis represents a general biological property shared by diverse network-oriented therapeutic interventions or whether distinct classes of mitochondrial modulators achieve network stabilization through different regulatory mechanisms.

Sixth, the concept of mitochondrial homeostasis itself will require continued conceptual and experimental refinement. Although the framework proposed in this review integrates mitochondrial bioenergetics, metabolic flexibility, redox regulation, proteostasis, MQC, and adaptive signaling into a unified systems-level model, additional regulatory layers governing mitochondrial adaptation will likely emerge as analytical technologies continue to evolve [34]. Future advances in functional multi-omics, spatial and single-cell proteomics, computational systems biology, artificial intelligence-assisted network reconstruction, and quantitative network modeling are expected to further refine our understanding of the mechanisms that coordinate MCBNs. Accordingly, mitochondrial homeostasis should be regarded as a dynamic and evolving biological property whose mechanistic definition will continue to improve as new experimental evidence becomes available [35].

Seventh, the quantitative characterization of MCBNs remains a major methodological challenge. Although advances in proteomics, functional multi-omics, and computational biology have substantially improved our ability to identify molecular components and regulatory pathways, robust computational frameworks capable of reconstructing network architecture, identifying regulatory hubs, quantifying network integrity, and measuring the organizational state of mitochondrial homeostasis are still under active development. The successful translation of the proposed framework into precision mitochondrial medicine will therefore depend on the development and validation of standardized computational approaches that generate reproducible, biologically interpretable, and clinically meaningful network signatures. Continued advances in systems biology, artificial intelligence-assisted network analysis, and quantitative computational modeling are expected to play a central role in achieving these objectives. Accordingly, future investigations should focus not only on identifying additional molecular components of MCBNs, but also on elucidating how their coordinated interactions generate the emergent properties underlying mitochondrial homeostasis.

From this perspective, the Mitochondrial Homeostasis Hypothesis should be regarded as an evolving systems biology framework that integrates comparative proteomics, functional multi-omics, experimental physiology, computational biology, and network science into a unified strategy for investigating metabolic disease. Continued experimental validation, methodological refinement, and interdisciplinary collaboration will ultimately determine its value for advancing our understanding of mitochondrial adaptation and for translating systems-level biological knowledge into precision mitochondrial medicine.

Despite the limitations discussed above, the remarkable convergence of proteomic, bioenergetic, redox, metabolic, signaling, and physiological evidence supports continued investigation of metabolic disease as a disorder of coordinated MCBNs rather than as a collection of isolated molecular abnormalities. Within this framework, preservation or restoration of mitochondrial homeostasis emerges not only as a measurable systems-level biological phenotype, but also as a promising conceptual foundation for the future prevention, diagnosis, and treatment of obesity, T2D, and other chronic diseases associated with mitochondrial dysfunction.

## 12. Conclusions

Obesity, T2D, and hypercaloric diet-induced metabolic dysfunction are complex multifactorial disorders characterized by profound disturbances in energy metabolism, oxidative stress, chronic inflammation, and tissue remodeling. Although these pathological processes have traditionally been investigated as largely independent mechanisms, the evidence reviewed throughout this manuscript supports a systems-level perspective in which they represent highly interconnected manifestations of a common biological disturbance centered on mitochondrial dysfunction [3,4]. This integrative view provides a conceptual framework for understanding metabolic disease as the consequence of disrupted coordination among adaptive biological processes rather than the cumulative effect of isolated molecular abnormalities.

Comparative proteomic studies reviewed throughout this manuscript have substantially advanced our understanding of metabolic disease by demonstrating that mitochondrial dysfunction involves coordinated remodeling of multiple interconnected biological processes rather than isolated alterations in individual proteins or signaling pathways. Across diverse experimental models and tissues, proteomic analyses consistently identify highly conserved changes affecting OXPHOS, FAO, TCA cycle activity, redox regulation, mitochondrial proteostasis, and adaptive signaling networks. The remarkable convergence of these findings across metabolically distinct organs-including the liver, heart, kidney, pancreas, and brain-supports the existence of conserved organizational principles governing mitochondrial adaptation during metabolic stress and provides the experimental foundation for the MCBN framework proposed in this review. Collectively, these observations support a fundamental shift in our understanding of mitochondrial biology. Rather than functioning solely as energy-producing organelles, mitochondria emerge as dynamic regulatory hubs that coordinate bioenergetics, substrate utilization, redox homeostasis, proteostasis, inflammatory signaling, and tissue adaptation through highly interconnected MCBNs. Within this framework, mitochondrial dysfunction is more appropriately interpreted as a progressive disruption of coordinated network organization, whereas mitochondrial homeostasis represents an emergent systems-level property arising from the dynamic interactions among conserved functional modules rather than from the activity of individual molecular pathways.

The collective evidence generated by our research group and corroborated by numerous independent studies further demonstrates that curcumin provides a representative experimental example of coordinated mitochondrial network modulation during metabolic stress. Across multiple tissues and experimental models, curcumin consistently preserves mitochondrial bioenergetics, supports FAO, attenuates oxidative stress, suppresses inflammatory signaling, limits fibrotic remodeling, and promotes adaptive mitochondrial responses [15,17]. Importantly, these protective effects occur in parallel with coordinated regulation of interconnected adaptive pathways, including the AMPK–SIRT1–PGC-1α axis, PPARα, PPARγ, and Nrf2, together with attenuation of maladaptive inflammatory signaling mediated by NF-κB and related regulatory networks. Rather than representing isolated molecular effects, these observations support the concept that curcumin acts as a representative network-modulating intervention capable of preserving the coordinated organization of MCBNs.

A central contribution of this review is the development of the MCBN framework, which reconceptualizes mitochondria not simply as energy-producing organelles but as dynamic regulatory hubs that coordinate bioenergetics, substrate utilization, redox homeostasis, proteostasis, inflammatory signaling, MQC, and tissue adaptation through highly interconnected biological networks. Within this systems-level framework, mitochondrial homeostasis emerges as an emergent biological property arising from the coordinated interactions among conserved mitochondrial functional modules rather than from the activity of individual molecular pathways. This perspective provides a unifying organizational model that integrates the diverse molecular, proteomic, physiological, and functional observations reviewed throughout this manuscript into a coherent systems biology framework for understanding metabolic adaptation and disease progression.

Building on the MCBN framework, this review proposes the Mitochondrial Homeostasis Hypothesis as a testable systems biology framework for understanding metabolic adaptation during obesity, T2D, and hypercaloric diet-induced metabolic dysfunction. The hypothesis proposes that preservation or restoration of mitochondrial homeostasis depends on the coordinated regulation of MCBNs governing OXPHOS, FAO, redox homeostasis, proteostasis, MQC, and adaptive signaling. Within this framework, representative network-modulating interventions, including curcumin, may promote tissue resilience by preserving the functional organization and adaptive capacity of these interconnected biological networks rather than by targeting individual molecular pathways. More broadly, the Mitochondrial Homeostasis Hypothesis provides a unified conceptual model linking molecular regulation, mitochondrial adaptation, systems biology, and translational medicine while generating experimentally testable predictions for future investigation.

The continued development of comparative proteomics, functional proteomics, redox proteomics, acetylomics, spatial and single-cell proteomics, integrated multi-omics, and artificial intelligence-assisted network analysis provides an unprecedented opportunity to experimentally validate and refine the Mitochondrial Homeostasis Hypothesis. By enabling quantitative characterization of MCBNs, these complementary approaches will facilitate the identification of network-based biomarkers of mitochondrial homeostasis, improve mitochondrial phenotyping, and support reconstruction of patient-specific network signatures. Collectively, these advances establish the methodological foundation for translating systems-level mitochondrial biology into network-guided precision mitochondrial medicine, where therapeutic strategies may be guided by the organizational state and adaptive capacity of mitochondrial biological networks rather than by isolated molecular targets.

In summary, the evidence reviewed throughout this manuscript supports a paradigm shift in which metabolic disease is viewed not as the consequence of isolated molecular abnormalities, but as a progressive disruption of the coordinated organization of MCBNs. Within this systems-level perspective, mitochondrial homeostasis emerges as a measurable and dynamic biological property that integrates bioenergetics, metabolic flexibility, redox regulation, proteostasis, MQC, and adaptive signaling into a unified framework of mitochondrial adaptation. The Mitochondrial Homeostasis Hypothesis provides a testable conceptual model that links comparative proteomics, functional multi-omics, systems biology, and network medicine while generating experimentally verifiable predictions for future investigation. As this framework continues to be refined through interdisciplinary research, it has the potential to reshape our understanding of obesity, T2D, and related metabolic disorders, establishing coordinated preservation of mitochondrial homeostasis as a promising foundation for future network-guided precision mitochondrial medicine.

## Figures and Tables

**Table 2:** Representative mitochondrial proteins and biological modules recurrently altered in obesity, T2D and hypercaloric diet-induced metabolic dysfunction.

Biological Module	Representative Proteins	Principal Function	Typical Alteration in Metabolic Disease	Effect of Curcumin
Oxidative Phosphorylation (OXPHOS)	ATP5A1, ATP5B, NDUFS1, SDHA, UQCRC1, COX5A	ATP production and electron transport	Reduced expression and impaired respiratory efficiency	Partial restoration of mitochondrial bioenergetics
Fatty Acid Oxidation (FAO)	CPT1A, ACADM (MCAD), ACADVL (VLCAD), HADHA	Lipid utilization and energy production	Reduced lipid oxidation and metabolic inflexibility	Improved fatty acid utilization
TCA Cycle	PDHA1, SUCLG2, PCX, ACO2	Central carbon metabolism	Impaired substrate oxidation	Enhanced metabolic adaptation
Redox Regulation	PRDX1, PRDX3, TXN, GPX1, SOD2	Antioxidant defense and redox signaling	Increased oxidative stress and ROS accumulation	Enhanced antioxidant defenses
Proteostasis	HSPD1, HSPA9, CLPP, LONP1	Protein folding, quality control and mitochondrial maintenance	Proteostasis disruption and mitochondrial stress	Improved protein quality control
Stress Adaptation	HSP family proteins, UPR^mt^-associated proteins	Cellular resilience and adaptive responses	Reduced adaptive capacity	Enhanced stress tolerance

Abbreviations: CPT1A, carnitine palmitoyltransferase 1A; FAO, fatty acid oxidation; GPX1, glutathione peroxidase 1; OXPHOS, oxidative phosphorylation; ROS, reactive oxygen species; TCA, tricarboxylic acid cycle; TXN, thioredoxin.

**Table 3:** Proteomic studies from our research group supporting the concept of mitochondrial-centered biological networks in metabolic disease.

Tissue	Experimental Model	Proteomic Approach	Principal Findings	Relevance to MCBNs	Reference
Liver	High-fat diet-induced obesity	Biochemical and mitochondrial analyses	Reduced oxidative stress and lipid peroxidation; improved mitochondrial function after curcumin treatment	Redox regulation and mitochondrial preservation	Martínez-Morúa et al., 2013 [58]
Liver and Kidney	db/db diabetic mice	Mitochondrial functional assays	Restoration of mitochondrial respiration and reduction of oxidative damage by curcumin	Mitochondrial homeostasis	Soto-Urquieta et al., 2014 [59]
Liver	db/db diabetic mice	Molecular signaling analysis	Curcumin modulated AMPK, NF-κB and PPARγ pathways	Metabolic signaling networks	Jiménez-Flores et al., 2014 [60]
Pancreas	db/db diabetic mice	2D-PAGE/MALDI-TOF proteomics	Altered proteins associated with oxidative stress, metabolism and cellular adaptation	Proteostasis and metabolic adaptation	Pérez-Vázquez et al., 2014 [61]
Liver	db/db diabetic mice	Comparative proteomics	Differential expression of proteins involved in carbohydrate and lipid metabolism	Metabolic network remodeling	Guzmán-Flores et al., 2016 [50]
Liver	20-week-old db/db diabetic mice	Proteomics and bioinformatics (GO, KEGG, STRING)	Enrichment of mitochondrial proteins involved in TCA cycle, redox regulation and energy metabolism	Evidence supporting mitochondrial-centered biological networks	Guzmán-Flores et al., 2018 [51]
Brain	Obesity and diabetes models	Biochemical and molecular analyses	Increased BDNF and reduced oxidative damage following curcumin treatment	Neuroprotection and redox adaptation	Franco-Robles et al., 2014 [62]

Abbreviations: AMPK, AMP-activated protein kinase; BDNF, brain-derived neurotrophic factor; GO, Gene Ontology; KEGG, Kyoto Encyclopedia of Genes and Genomes; MCBNs, mitochondrial-centered biological networks; NF-κB, nuclear factor kappa B; PPARγ, peroxisome proliferator-activated receptor gamma.

**Table 4:** Signaling pathways modulated by curcumin and their relationship with mitochondrial homeostasis.

Signaling Pathway	Physiological Function	Alteration in Obesity and T2D	Effect of Curcumin	Consequences for Mitochondrial Homeostasis	Representative References
AMPK	Cellular energy sensing; regulation of catabolic and anabolic pathways	Reduced activation contributes to metabolic inflexibility, lipid accumulation and impaired mitochondrial biogenesis	Activates AMPK signaling and improves energy metabolism	Enhances mitochondrial biogenesis, fatty acid oxidation and metabolic adaptation	Kim et al., 2009 [86]; Jiménez-Flores et al., 2014 [60]
SIRT1	NAD^+^-dependent deacetylase regulating mitochondrial metabolism and stress responses	Reduced activity impairs mitochondrial function and adaptive responses	Increases SIRT1 activity and downstream signaling	Promotes mitochondrial maintenance, stress resistance and metabolic flexibility	Rodgers et al., 2005 [108]; Hou et al., 2024 [87]
PGC-1α	Master regulator of mitochondrial biogenesis and oxidative metabolism	Reduced expression is associated with impaired oxidative capacity	Upregulates PGC-1α expression and activity	Increases mitochondrial content, respiration and oxidative metabolism	Puigserver and Spiegelman, 2003 [109]; Hou et al., 2024 [87]
PPARα	Regulation of fatty acid transport and β-oxidation	Reduced activity contributes to lipid accumulation and lipotoxicity	Enhances PPARα signaling	Improves fatty acid utilization and mitochondrial substrate oxidation	Kersten, 2014 [89]; Jiménez-Flores et al., 2014 [60]
PPARγ	Regulation of insulin sensitivity, lipid storage and glucose metabolism	Dysregulation contributes to insulin resistance and chronic inflammation	Modulates PPARγ activity and improves insulin sensitivity	Supports metabolic homeostasis and reduces inflammatory stress	Evans et al., 2004 [90]; Jiménez-Flores et al., 2014 [60]
Nrf2	Master regulator of antioxidant and cytoprotective responses	Impaired activation increases oxidative stress and cellular vulnerability	Activates Nrf2-dependent antioxidant pathways	Preserves redox homeostasis and limits oxidative damage to mitochondrial proteins	Ma, 2013 [93]; Scapagnini et al., 2011 [95]
NF-κB	Central mediator of inflammatory signaling	Chronic activation promotes cytokine production, oxidative stress and tissue injury	Suppresses NF-κB activation and inflammatory responses	Reduces mitochondrial stress and prevents network deterioration	Reuter et al., 2010 [106]; Jiménez-Flores et al., 2014 [60]
NLRP3 Inflammasome	Innate immune sensor activated by metabolic and oxidative stress	Persistent activation contributes to chronic inflammation and tissue remodeling	Attenuates inflammasome activation and cytokine release	Limits inflammation-mediated mitochondrial dysfunction	Kelley et al., 2019 [110]; Kunnumakkara et al., 2017 [17]
TGF-β	Regulation of extracellular matrix deposition and fibrosis	Excessive activation promotes fibrotic remodeling and organ dysfunction	Inhibits TGF-β-mediated fibrotic signaling	Preserves tissue architecture and mitochondrial network integrity	Wynn and Ramalingam, 2012 [77]; Frangogiannis, 2020 [78]

Abbreviations: AMPK, AMP-activated protein kinase; MCBNs, mitochondrial-centered biological networks; NF-κB, nuclear factor kappa B; NLRP3, NOD-like receptor family pyrin domain containing 3; Nrf2, nuclear factor erythroid 2-related factor 2; PGC-1α, peroxisome proliferator-activated receptor gamma coactivator 1 alpha; PPAR, peroxisome proliferator-activated receptor; SIRT1, sirtuin 1; T2D, type 2 diabetes mellitus; TGF-β, transforming growth factor beta.

**Table 5:** Emerging proteomic technologies and their potential contribution to precision mitochondrial medicine.

Technology	Molecular Information Obtained	Resolution	Potential Contribution to Mitochondrial Medicine
Conventional Proteomics	Global protein abundance	Tissue level	Identification of disease-associated protein signatures
Redox Proteomics	Oxidative post-translational modifications	Protein-specific	Characterization of redox-sensitive mitochondrial proteins
Phosphoproteomics	Phosphorylation-dependent signaling events	Site-specific	Identification of adaptive and maladaptive signaling pathways
Acetylomics	Lysine acetylation profiles	Site-specific	Evaluation of SIRT1-regulated mitochondrial adaptation
Spatial Proteomics	Protein localization within tissue architecture	Cellular and subcellular	Identification of region-specific mitochondrial remodeling
Single-Cell Proteomics	Protein expression in individual cells	Single-cell	Characterization of cellular heterogeneity and vulnerable populations
Multi-Omics Integration	Integration of transcriptomic, proteomic, metabolomic, lipidomic and epigenomic data	Systems level	Reconstruction of mitochondrial-centered biological networks and patient stratification
Artificial Intelligence and Network Modeling	Predictive network analysis	Systems level	Identification of molecular signatures and personalized therapeutic targets

## Data Availability

No new data were created or analyzed in this study. Data sharing is not applicable to this article.

## Abbreviations

AMPK, AMP-activated protein kinase; FAO, fatty acid oxidation; MCBNs, mitochondrial-centered biological networks; NF-κB, nuclear factor kappa B; Nrf2, nuclear factor erythroid 2-related factor 2; OXPHOS, oxidative phosphorylation; PGC-1α, peroxisome proliferator-activated receptor gamma coactivator 1 alpha; PPAR, peroxisome proliferator-activated receptor; ROS, reactive oxygen species; SIRT1, sirtuin 1; T2D, type 2 diabetes; TCA, tricarboxylic acid.
